# Revisiting the grammaticalization of future *be going to*: A corpus-based approach

**DOI:** 10.1371/journal.pone.0352674

**Published:** 2026-07-24

**Authors:** Junhui Wu, Zerui Dai

**Affiliations:** School of Foreign Studies, Changsha University of Science & Technology, Changsha, Hunan, China; Bahir Dar University, ETHIOPIA

## Abstract

Through the quantification of frequency, existing corpus-based quantitative studies on grammaticalization offer a new direction for empirical investigation. However, most existing studies focus on general frequencies, failing to elucidate the relationship between frequency and grammaticalization. The proposition of critical frequency can reveal the relationship between frequency and grammaticalization and confirm the “threshold” that triggers grammaticalization. The present study explores the critical frequency (i.e., synchronic intensity and diachronic thickness) of future *be going to* and clarifies the relationship between its critical frequency and general frequency based on the corpora of EEBO, Google Books and COCA. The corpora are large enough for tracing the development of the fully grammaticalized *be going to*. Furthermore, the results retrieved from them can be mutually verified, thereby ensuring the validity of the present study. Quantitative analyses, including Chi-square test and linear regression test, are performed on the retrieved results. The findings include: (1) the diachronic thickness of future *be going to* lasts for 110 years (i.e., 1580–1690) and the synchronic intensity amounts to 0.81 per million words; (2) The critical frequency and the general frequency of future *be going to* are closely correlated. The present study confirms the threshold for triggering the grammaticalization of future *be going to* and elucidates the relationship between its critical frequency and general frequency. This study provides insights for future quantitative research on grammaticalization.

## Introduction

It is accepted that the *be going to + N* construction is the starting point of the grammaticalization of future *be going to* [1–11]. For example:

(1) a. Tomorrow I*’m going to London* for a few days. (COCA_WEB)
b. I*’m going to visit* one of the boot camps here in Georgia tomorrow. (COCA_NEWS)
c. There was no way he *was going to love* this woman. (COCA_FIC)

According to Pérez [1] and Yaguchi [11], the preposition *to* in (1a) is reanalyzed as the infinitive marker *to* in (1b) and (1c), and the full grammaticalization of future *be going to* is realized by analogy. If it were the case, how would the preposition *to* as in (1a) be shifted to the infinitive marker *to* as in (1b) by reanalysis?

Grammaticalization is instigated by frequency [12] and high frequency is the prerequisite of grammaticalization [13]. The combination of language units is the result of higher frequency of use [5,12,14–18]. Frequency theory not only underscores the crucial role of frequency in triggering grammaticalization, but also provides a quantitative research orientation for the study of grammaticalization. However, lower frequency can also instigate grammaticalization. Heine et al. [13] and Heine & Kuteva [5] further hold that high frequency does not necessarily lead to grammaticalization. The validity of frequency theory for grammaticalization has been challenged both theoretically and empirically. The reason lies in the fact that pragmatic inference taken as the mechanism of grammaticalization operates only in specific context. Frequency theory posits that frequency facilitates grammaticalization, but it fails to further strictly distinguish contexts that can trigger grammaticalization from those that cannot. Therefore, its conclusion is based on general frequency. Regarding the interrelationship between frequency and grammaticalization, Heine [19] and Diewald [20] proposed the concept of critical frequency. The context in which critical frequency arises is termed as critical context by Diewald [20]. With ambiguity as its distinctive features, critical context provides room for the grammaticalization of grammaticalizing items. Diewald’s critical context is characterized by “multiple structural and semantic ambiguities” and can invite “several alternative interpretations” with new grammatical meaning contained [20]. Grammaticalization will be triggered providing that the critical frequency accumulates a considerable diachronic thickness (i.e., the persistent occurrence of critical context instances of the grammaticalizing items across continuous synchronic planes leads to the repetition of similar pragmatic inference diachronically, thus forming the diachronic thickness of pragmatic inference) and reaches a certain synchronic intensity (i.e., the high frequency of critical context instances of grammaticalizing items at certain synchronic plane leads to the repetition of similar pragmatic inference, thus forming the synchronic intensity of pragmatic inference) [21]. As only the critical frequency can facilitate the fulfillment of pragmatic inference, the study of the critical frequency of the grammaticalizing items can be regarded as the integration study of frequency and that of mechanism of grammaticalization. That is, the critical frequency of grammaticalization bridges the study of frequency to that of mechanism of grammaticalization. The exploration of the critical frequency of the grammaticalizing items will reveal the true “threshold” of grammaticalization.

The construction of *be going to* serves as a typical example frequently referred to in the exploration of grammaticalization. However, most of the occurrences of the construction only serve as examples for the illustration of grammaticalization theory. The exploration of the construction itself needs further attention. The current study of the grammaticalization of *be going to* can be categorized into the following aspects: (1) the motivations of the grammaticalization of *be going to* [2,14,22,23]; (2) the mechanisms of the grammaticalization of *be going to* [24,25]; (3) the starting point and ending time of the grammaticalization of *be going to* [26–29]; (4) the grammatical category of *be going to* [25,29,30]; (5) the geographical and stylistic distribution of the grammaticalization of *be going to* [3,31,32]. All of the above studies are based on general frequency instead of critical frequency. Until now, there has been no study of the critical frequency of the grammaticalization of *be going to*. The purpose of this study is to investigate the critical frequency of future *be going to* and to reveal the relationship between its critical frequency and general frequency. For this purpose, this study will be conducted with large-size historical corpora. We shall first review grammaticalization and then introduce the corpora selection and data collection, and present the discussion and the findings of the research.

## Grammaticalization

### Context

Grammaticalization is triggered in context [20]. The study of context is a prerequisite for the exploration of the relationship between grammaticalization and frequency. Heine [33] and Traugott [34] pointed out the importance of morphosyntactic and pragmatic contexts in grammaticalization. According to Peng [21], pragmatic inference as the mechanism of grammaticalization operates only in specific contexts. If pragmatic inference does not occur, the highest frequency will not trigger grammaticalization.

Heine [19] identified three types of contexts: bridging context, switch context and conventionalization, and thereby classified the grammaticalization process into four stages: initial stage, bridging context stage, switch context stage and conventionalization stage. Diewald [20] also proposed three types of contexts: untypical context, critical context and isolating context. Critical context bears the critical characteristics for evoking the emergence of the target meaning, i.e., the pragmatic inference condition. The repetitive occurrences of pragmatic inference will lead to the entrenchment of the target meaning. The critical context is characterized by the potential to evoke ambiguity. The function of critical context is to provide room for the change of the grammaticalizing items.

Based on Heine [19] and Diewald [20], we recategorize three types of contexts in the present study for the investigation of the grammaticalization process of future *be going to*: initial stage, critical context and isolating context. Initial stage is equal to Heine’s [19] initial stage of grammaticalization and overlaps with Diewald’s [20] untypical context. Critical context covers Heine’s [19] bridging context and switch context and Diewald’s [20] critical context. Isolating context covers both conventionalization by Heine [19] and isolating context by Diewald [20].

The initial stage constitutes the preconditions for later grammaticalization. This stage would keep semantically and structurally stable without ambiguous reading. Once ambiguities arise, grammaticalization will be triggered in the critical context. Therefore, the initial stage merely provides a necessary precondition for grammaticalization instead of initiating grammaticalization.

It is only in critical contexts that the pragmatic inference can be fulfilled. In the critical context, semantic and structural ambiguities may appear, which will trigger grammaticalization. It will lead to several alternative interpretations. According to Heine [19], while the target meaning is the one most likely to be inferred, it is still “cancellable”, that is, an interpretation in terms of the source meaning cannot be ruled out. A linguistic form may be associated with a number of different critical contexts. For example, in the *be going to +V* construction, *V* can be any verb as long as it can meet the requirement of the critical context.

The isolating context is incompatible with some salient properties of the source meaning. In the isolating context, the target meaning is favored and isolated as a distinct meaning from the source meaning. Through the entrenchment of favoritism, the status of the target meaning is firmly established. The interpretation of the target meaning no longer needs to be supported by context; rather it has been freed from the contextual constraints that gave rise to it [19] and turns into the “normal” or “inherent” or “usual” or “semantic” meaning [26]. Now it can be used in all types of contexts.

It should be noted that there are no clear-cut boundaries between these three stages. Any attempt to divide them is arbitrary, and even the critical context can be refined by finer scales.

### Critical context and critical frequency

Of the critical context and untypical context, only critical context may cause pragmatic inference, which has an effect on grammaticalization. Based on critical context and untypical context, Peng [21] distinguished two types of frequencies of the grammaticalizing item, i.e., critical frequency and non-critical frequency. Critical frequency is independent of the general frequency in that the increase of the former may (but not necessarily) accompany the increase of the latter. Peng [21] further hypothesized that the premise of grammaticalization is the repetition of similar pragmatic inference caused by the constant occurrences of critical context instances.

To illustrate the validity of critical frequency, Peng [21] investigated the critical frequency of some morphemes and constructions in Chinese. The former includes “shi (时)” with its grammaticalization from temporal noun to hypothetical particle. The latter covers the competition between two non-structural strings of “yin (因)+er (而)” and “yin (因)+yi (以)”. By determining the critical frequency as well as the general frequency of these morphemes and constructions, Peng [21] conducted a preliminary exploration of the critical frequency in grammaticalization. Peng [35] further explored two issues about critical frequency: (1) whether there exists a positive correlation between general frequency and critical frequency; (2) what the minimum critical frequency required for pragmatic implicature to become conventionalized is. These pioneering studies have undertaken preliminary explorations of critical frequency through case studies of Chinese morphemes and constructions. However, the universality of critical frequency requires further typological evidence.

### Competition between candidate future expressions

In the *be going to* expression, the progressive generally means action in process. The notion of movement in space can be extended to that in time by metaphor. *be going* is accordingly interpreted as a purposive expression relevant to the time referred to in the clause and likely to be imminent [1,25,36]. The candidates for such a usage are not confined to *go*. According to Pérez [1] and Bybee et al. [2], *go* and *come* are the most common movement verbs susceptible to be grammaticalized to a future marker. The reason is that both verbs are unmarked for the manner of movement. Actually, *leave* can also be added to the list as a candidate for a future marker. The question is why only *go* wins the competition.

## Grammaticalization process of *be going to*

The *be going to* construction in (1a) can be comparable with the *be arriving at* construction as in *I am arriving at London*; whereas the *be going to* construction in (1b) is equivalent to *be going in order to* as in *I’m going in order to visit one of the boot camps here in Georgia tomorrow*. Hopper & Traugott [25] referred to this infinitive marker *to* as “purposive *to*”. Hopper & Traugott [25] also considered that *be going to* can be grammaticalized only in very “local contexts”. It is the *be going to + V* construction rather than the *be going to + N* construction that is the starting point of grammaticalization of future *be going to*. Some researches (Poplack & Tagliamonte [3], Traugott & Dasher [27]) touch upon the issue of when the first valid example of *be going to + V* appeared. Both of them refer to the same example as the first plausible one for *be going to+ V*, which appeared in the year of 1482. Considering the variants of Middle English, *be goyng to* is the early form of *be going to* [33]. In EEBO, the frequency of *be goyng to* is retrieved by the SQ of “[vb*] goyng to [v*]”. With consideration of the variants of *be going to* (i.e., *be goyng(e) to*), the first valid example appeared in 1477 in EEBO corpus with the search queries of “[vb*] goyng to [v*]” and “[vb*] goyng(e) to [v*]” respectively. The first valid example of *be going to* + *V* appeared in the year 1477 instead of 1482.

This study will investigate the critical frequency and the general frequency of future *be going to*. The hypothesis underlying the research reported in this paper is that the *go and V* construction has the potential to shift to the purposive *go to V* construction and that any movement verb not marked for the specific manner can be a candidate for the competition for future expression.

Based on the schema of the development of auxiliary *be going to* and the revised schema proposed in Hopper & Traugott [25] as well as the diachronic change from *go and V* to *go to V*, this section puts forward the whole grammaticalization process of *be going to* can be classified into 6 stages. The *go and V* construction can be considered as the first stage. It has the potential to shift to the *go to V* construction, which can be regarded as a case of grammaticalization, with the infinitive marker *to* expressing the purposive meaning. The third stage *go + to V* is demonstrated by any tense of *go* as the directional verb with a purposive clause. By reanalysis, the relationship between *to* and *V* becomes closer, while that between *go* and purposive clause *to V* seems to be looser, which offers potential for the change (including the progressive aspect) of *go*. The fourth stage is manifested as the progressive with the directional verb and a purposive clause, *be going (to V).* As mentioned above, at least three candidate directional verbs compete for the future expression. It is *go* that has finally won the competition. The fifth stage is demonstrated by the future auxiliary with a verb *(be going to) V*. This stage is the reanalysis of the fourth stage. The fifth stage can be further divided into substages by analogy: (1) as the starting point for the grammaticalization of *be going to*, the “*be going to +* action verb” construction is the initial stage of grammaticalization; (2) the “*be going to* + abstract verb” construction is the critical context in which ambiguity arises, and (3) the “*be going to* + mental or stative verb” construction is the isolating context of future *be going to*. The sixth stage is the consequence of the reanalysis of the future *be going to* to a single morpheme *gonna*. All these stages coexist in modern English, which Hopper [37] referred to as “layers”. Here are some examples for the six stages:

Stage 1: go and V
(2) Why don’t you *go and get* us another round of those specials? (COCA_FIC)
Stage 2: go to V
(3) I *went to see* him in the hospital. (COCA_FIC)
Stage 3: go (to V)
(4) I *went to see* the dog. (COCA_TV)
(5) I couldn’t *go* back *to sleep* after my nightmare. (COCA_TV)
Stage 4: be going (to V)
(6) I thought you *were going* straight *to work*. (COCA_MOV)
Stage 5: (be going to) V
(7) We*’re going to find out* more details about them. (COCA_SPOK)
(8) I *am going to see* The Dark Knight Rises. (COCA_BLOG)
(9) She knew she *wasn’t going to like* what he had to say next. (COCA_FIC)
Stage 6: (be) gonna V
(10) I’*m gon na talk to* Bobby. (COCA_TV)

In Example (2), the two verbs of *go* and *get* serve as predicates. It seems the two predicates have the same syntactic status. The only difference lies in their sequence, i.e., “you must first go, then you can get.”, not vice versa. Therefore, the two verbs are in “asyndetic coordination” [30]. Lord [38] put that, “in many languages, the verbs in a serial construction tend to refer to sub-parts of a single overall event, and the second verb phrase is typically a further development, result, or goal of the first verb phrase.” The independence of *get* decreases to avoid the competition between the two verbs of *go* and *get*.

Therefore, in Example (3) the two verbs of *went* and *see* expressing sequential actions have different syntactic status. *Went* serves as the predicate while *see* (with *to* functioning as the infinitive marker) serves as adverbial of purpose. The syntactic hierarchy between the two actions are clearly demonstrated.

From Stage 2 to Stage 3, the change is fulfilled by syntactic reanalysis. The closeness between *to* and *V* as well as the looseness between *go* and purposive clause *to V* can be discerned in examples. As reanalysis is not directly observable in surface manifestation [25], it seems that Example (4) is the same as Example (3). But in Example (5), the looseness between *go* and purposive clause *to sleep* is demonstrated by the appearance of the adverb *back*. That is to say, *go* and *to sleep* are so loosely connected that some elements as adverbs can be inserted between them.

Stage 4 is demonstrated as the progressive of *go* and a purposive clause. In Example (6), the relationship between them is so loose that the adverb of *straight* can be inserted. At this stage, *go* can only be interpreted as a movement in space due to the appearance of *straight*.

From Stage 4 to Stage 5, the change is fulfilled by syntactic reanalysis. In Stage 5, *go* can be extended from space to time by means of metaphor. *Be going to* has two possible interpretations of movement in space and future in time. As the ambiguity arises, the critical context for the grammaticalization of *be going to* comes into being. In Example (7), *be going to* is followed by the phrase “*find out*”, which indicates a concrete action, and *be going to* here has two interpretations. The first one is “We are moving from one place to another with the aim of finding out more details” while the second one is “We will find out more details in the future.” Thus, ambiguous interpretations arise, which forms the critical context for the grammaticalization for *be going to*. In Example (8) and (9), *be going to* is followed by mental verbs of *see* and *like* [39], which is the demonstration of the full grammaticalization of future *be going to* [40]. In Example (8) and (9), *be going to* has only one interpretation as the future marker without any ambiguity. After Stage 5, the status of future marker *be going to* is firmly established.

Stage 6 sees the phonological erosion of *be going to*, which is a kind of manifestations of grammaticalization. What’s more, *gonna* appears more frequently in spoken form, as can be seen in Example (10).

## Methodology

### Corpora

In this study, we use the Early English Books Online (EEBO) (https://www.english-corpora.org/eebo/) and the Google Books Corpus (http://googlebooks.byu.edu/x.asp/).The former contains 755 million words from the 1470s to the 1690s. The default option of time span for each retrieval is 10 years. The starting point of the grammaticalization of future *be going to* can be assumed to begin from the mid 1400s [41–42] while the full grammaticalization of future *be going to* is realized in the late 17th century [29]. Therefore, the EEBO can be used for the investigation of the grammaticalization of future *be going to*. Google Books covers a time span of 500 years (1500–2000). It contains 200 billion words of data in both the American and British English datasets in total. It is large enough for tracing the development of the fully grammaticalized *be going to*.

### Data retrieval

Verbs that can occur in the grammaticalization process of future *be going to* can be categorized into three types. The first type of verbs cooccur in the *go and V* and *go to V* constructions. They may appear in the first stage of the grammaticalization process with no ambiguities arising. The second type of verbs are mental or stative verbs. They are the indicator of the full grammaticalization of *be going to*. According to Traugott [40] and Hopper & Traugott [25], the full grammaticalization of *be going to* “is evidenced when the following subject and/or the verb is incompatible with purposiveness”. The second type of verbs appear in the isolating context of the three contexts. The third type of verbs appear in the critical context. They have the potential to trigger grammaticalization of *be going to*. Their tendency to give rise to ambiguity may vary. For the comprehensiveness of the study, all the verbs which may give rise to ambiguity are taken into consideration without further considering the degree of ambiguity. The finer evaluation of the ambiguity they cause will call for further exploration. The total number of verbs appearing during the grammaticalization process of *be going to* minus the first two types of verbs leads to the third type of verbs.

In order to explore the relationship between the constructions of *go to V* and *go and V* diachronically and to confirm the words having potential to constitute the critical contexts (i.e., the contexts offering room for the occurrence of critical frequency) of *be going to*, the following search query is employed:

SQ1. [go] and|to [v*]

This search query can be described as any form of *go* followed by *and* or *to* and any form of a verb. The data retrieved in the Early Modern English (1500–1700) and the Late Modern English (1700–2000) for the two constructions using this search query are shown in Table 1.

**Table 1:** Data retrieved using SQ1 (per million words).

	1500-1700	1710-2000
go and V	137.66	381.69
go to V	348.41	1826.65
Total	486.07	2208.34

From Table 1, it can be seen that the frequencies of *go and V* construction during the periods of 1500–1700 and 1710–2000 are 137.66 pmw and 381.69 pmw respectively. And the frequencies of *go to V* construction during the two periods are 348.41 pmw and 1826.65 pmw accordingly. Data show that there are totally 1349 verbs (lemmas) appearing in the *go and V* construction and 1669 verbs (lemmas) in the *go to V* construction. Of these two groups of verbs, 763 occur in both the *go and V* and *go to V* constructions. Here are some examples:

(11) a. Isabella saw them talking together from the other door, and immediately *went and told* her mistress of it. (EEBO_1700)
b. And so it will be convenient to *go and take* them there. (EEBO_1698)
c. And that no church ought to be presumed to authorize her priests or bishops to *go and preach* the gospel after their private Sence or conscience. (EEBO_1687)
(12) a. So forth we *went to see* all the holy places in the city which were to be seen, except those in sepulchral sancta. (EEBO_1692)
b. With this determination she *went to visit* her the next day after her arrival. (EEBO_1652)
c. He would send him a golden ring, upon the receipt of which he would *go to meet* him. (EEBO_1700)

The candidates for such a usage are not confined to *go* only. Just as Pérez [1], Bybee et al. [2], Hopper & Traugott [25] mentioned, *go* as well as *come* are the most probable options for grammaticalization into a future marker. The reason lies in that both verbs are unmarked for the manner of movement, that is, they do not specify the details of the movement. They are in the basic-level category in cognition and are the most frequently used. Verbs meeting this standard are not confined to *go* and *come*. *Leave* can be added to the list. All of them are the candidates for the competition to undergo grammaticalization into a future marker, but it is only *go* that has won the competition. The frequencies of the three verbs are retrieved respectively in the corpus to serve as a demonstration. It should be noted that the frequencies are general frequencies instead of critical frequencies. To prove the competition between candidates for the future expression, the following search queries are used.

SQ2. [vb*] going to [v*]
SQ3. [vb*] coming to [v*]
SQ4. [vb*] leaving to [v*]

SQ2 can be described as *be going to* followed by an infinitive. SQ3 can be described as the structure of *be coming to* followed by an infinitive. SQ4 can be described as the structure of *be leaving to* followed by an infinitive. The result can explain which finally becomes the selected form of future expression. In EEBO, the normalized frequencies of the three structures are retrieved by the three search queries respectively. See Table 2 and examples (13) to (15):

**Table 2:** Frequencies of the three candidate future expressions in the EEBO (per million words).

	[vb*] going to [v*]	[vb*] coming to [v*]	[vb*] leaving to [v*]
1470s	0.00	0.00	0.00
1480s	0.00	0.00	0.00
1490s	0.00	0.00	0.00
1500s	0.00	0.00	0.00
1510s	0.00	0.00	0.00
1520s	0.00	0.33	0.00
1530s	0.14	0.00	0.00
1540s	0.11	0.00	0.00
1550s	0.00	0.00	0.00
1560s	0.25	0.12	0.00
1570s	0.33	0.07	0.00
1580s	0.59	0.03	0.00
1590s	0.71	0.08	0.00
1600s	1.00	0.02	0.00
1610s	0.61	0.00	0.00
1620s	0.96	0.00	0.00
1630s	1.35	0.05	0.00
1640s	2.76	0.30	0.00
1650s	3.91	0.99	0.00
1660s	4.17	1.10	0.02
1670s	7.44	1.31	0.00
1680s	7.26	1.20	0.00
1690s	10.56	1.20	0.01

(13) a. …he said, he *was going to* seek his bread. (EEBO_1691)
b. I *am going to* taste the sweets of repose, and *to* find tranquility at my own house. (EEBO_1692)
c. …that we *were going to* spread the gospel among the most savage nations in the world. (EEBO_1693)
(14) a. In his time some rebel Irishmen *were coming to* aid the Earl of Chester against the king. (EEBO_1690)
b. …he learnt that the holy prelat *was coming to* find him. (EEBO_1693)
c. …that he *is coming to* reform the disorders that this prince has caused in the churches of his dominions. (EEBO_1693)
(15) …, so have they not *been leaving to* distribute the same again in charitable uses… (EEBO_1668)

All of the three verbs have the potential to undergo the grammaticalization into a future marker. In the competition among the candidates, frequency serves as the crucial factor. From Table 2, it can be seen that the frequency of *go* overwhelms others, which makes it the only chosen form. The frequency of *leave* is too low to be further grammaticalized. As for *come*, out of the top 10 high-frequency words, 5 coincide with those from SQ2. They are *take* (with its frequency of 23), *see* (20), *meet* (15), *be* (14) and *make* (12). The fact means that there does exist critical context for the potential grammaticalization of *come* into a future marker. But the relative low frequency has not yet reached the threshold for the grammaticalization of *come*.

## Critical frequency of *be going to*

### Origin of the *be going to* + *V* construction

Quirk et al. [30] distinguished syndetic coordination and asyndetic coordination. Syndetic coordination is symmetrical, and hence the two elements are reversible, whereas asyndetic coordination is irreversible because the two elements are sequential. The displacement of the coordinate elements in asyndetic coordination will change the meaning [43]. Sentences a, b in Example (16) are both cases of asyndetic coordination.

(16) a. They *called and said* there was a box coming! (COCA_TV)
b. The New Milford police officer had *shot and killed* a fleeing suspect. (COCA_NEWS)

Asymmetric coordination is so commonly seen that it has become the unmarked organization [43]. The asymmetric “V_1_ and V_2_” construction is semantically equivalent to the purposive “V_1_ to V_2_” subordination in cases where V_1_ is a motion verb. For example:

(17) a. I need to *go and check* on Lily right now. (COCA_MOV)
b. So, I just wanted to *come and talk* to you about all this. (COCA_TV)
c. I’ll probably *stop and see* you before I go. (COCA_FIC)
(18) a. The Pig family *went to buy* the biggest pumpkin they could find. (COCA_FIC)
b. The Canadians *came to visit* the police department in Manchester last fall. (COCA_MAG)
c. He *stopped to look* at the paintings. (COCA_FIC)

**Fig 1:**
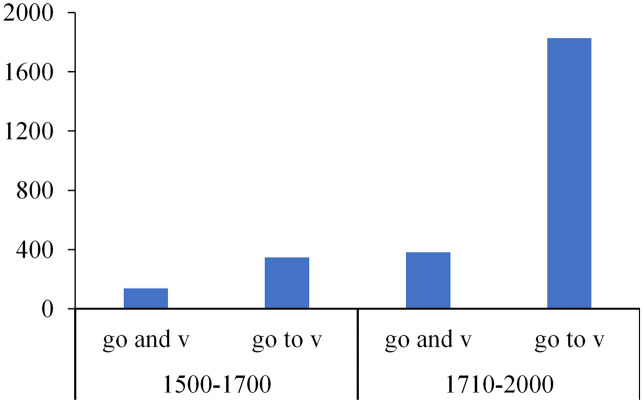
The go and V and go to V constructions in the two periods of Google Books Corpus.

In Example (17), V_1_ in “V_1_ and V_2_” construction are *go*, *come* and *stop* respectively, which are all motion verbs. Sentences in Example (17) are semantically corresponding to those in Example (18) accordingly. Synchronically, the asymmetric “V_1_ and V_2_” construction is semantically equivalent to the purposive “V_1_ to V_2_” subordination. Diachronically, the relationship between the two constructions requires further investigation. The question is which of the two constructions comes first in language evolution. In the following, we will trace the diachronic changes of the two constructions of *go and V* and *go to V* in Google Books Corpus. The normalized frequencies of the two constructions from Table 1 are shown in Fig 1.

Fig 1 shows that the normalized frequencies of both constructions are higher in the Late Modern English than those in the Early Modern English. Chi-square test is applied to determine the correlation between the two constructions. The data are from Table 1. The two groups of data are significantly differently distributed in the two sub-corpora (*χ*^2^ = 31.531, *df* = 1, *p* = 0.000 < 0.01) and the correlation strength is modest (*F* = 0.108). Comparatively, the *go to V* construction increases faster than the *go and V* construction, 5.24 times and 2.77 times faster than those in the first period respectively. This indicates that the asymmetrical *go and V* construction has the potential to shift to the purposive *go to V* construction.

The high-frequency words appearing in the constructions of *go and V* and *go to V* will be retrieved by SQ1 in EEBO. The top 200 high-frequency words in the two constructions will be listed in sequence respectively in Supporting Information Files. So the top 50, 100, and 200 high-frequency words cooccurring in the two constructions will be obtained. The results show that 28 words cooccur from the 50 high-frequency words in both constructions, 68 words cooccur from the 100 high-frequency words in both constructions, and 141 words cooccur from the 200 high-frequency words in both constructions. The ratios are 56%, 68% and 70.5% accordingly. The complete inventories of cooccurring words for two constructions are provided in Appendix A in S4 File.

When two verbs appear consecutively in the same sentence, the independence of one verb will decrease to avoid the competition between both verbs. The decreasing independence of the main verb is demonstrated by the defectiveness of the other verb. According to Lord [38], the change has been “along a grammaticalization continuum”. Therefore, the process from *go and V* to *go to V* is itself a case of grammaticalization.

### Synchronic intensity and diachronic thickness of *be going to*

In this section, we will investigate the critical frequency of *be going to* by its synchronic intensity and diachronic thickness.

Using SQ2, we collected 543 verbs appearing in the whole grammaticalization process of *be going to*. Of the 543 verbs, 381 occur in both the *go and V* and the *go to V* constructions. Verbs cooccur in the *go and V* and the *go to V* constructions will not cause ambiguity. The 381 verbs in *be going to* construction form the initial stage. All the 543 verbs appearing in the grammaticalization process of *be going to* minus the cooccurring 381 verbs result in 162 verbs.

According to Halliday & Matthiessen [39], among the 162 verbs, the three verbs of “recall, resolve, hate” belong to mental verbs. The three verbs in *be going to* construction form the isolating context. As mental verbs are the indicator of the full grammaticalization of *be going to* [29,40], the three verbs will be excluded from the list. The total frequencies of the 159 verbs (i.e., 162 minus 3) appearing in the *be going to V* construction form the critical context of the grammaticalization of future *be going to*. The complete inventory of these 159 verbs is listed in Appendix B in S5 File. See Table 3.

**Table 3:** General frequencies and critical frequencies of *be going to* (per million words).

	general frequency	critical frequency
1470s	0	0
1480s	0	0
1490s	0	0
1500s	0	0
1510s	0	0
1520s	0	0
1530s	0.14	0
1540s	0.11	0
1550s	0	0
1560s	0.25	0
1570s	0.22	0
1580s	0.59	0.09
1590s	0.71	0.12
1600s	0.87	0.09
1610s	0.54	0
1620s	0.88	0.03
1630s	1.19	0.11
1640s	2.46	0.18
1650s	3.48	0.36
1660s	3.61	0.41
1670s	6.43	0.66
1680s	6.20	0.61
1690s	8.73	0.81

**Fig 2:**
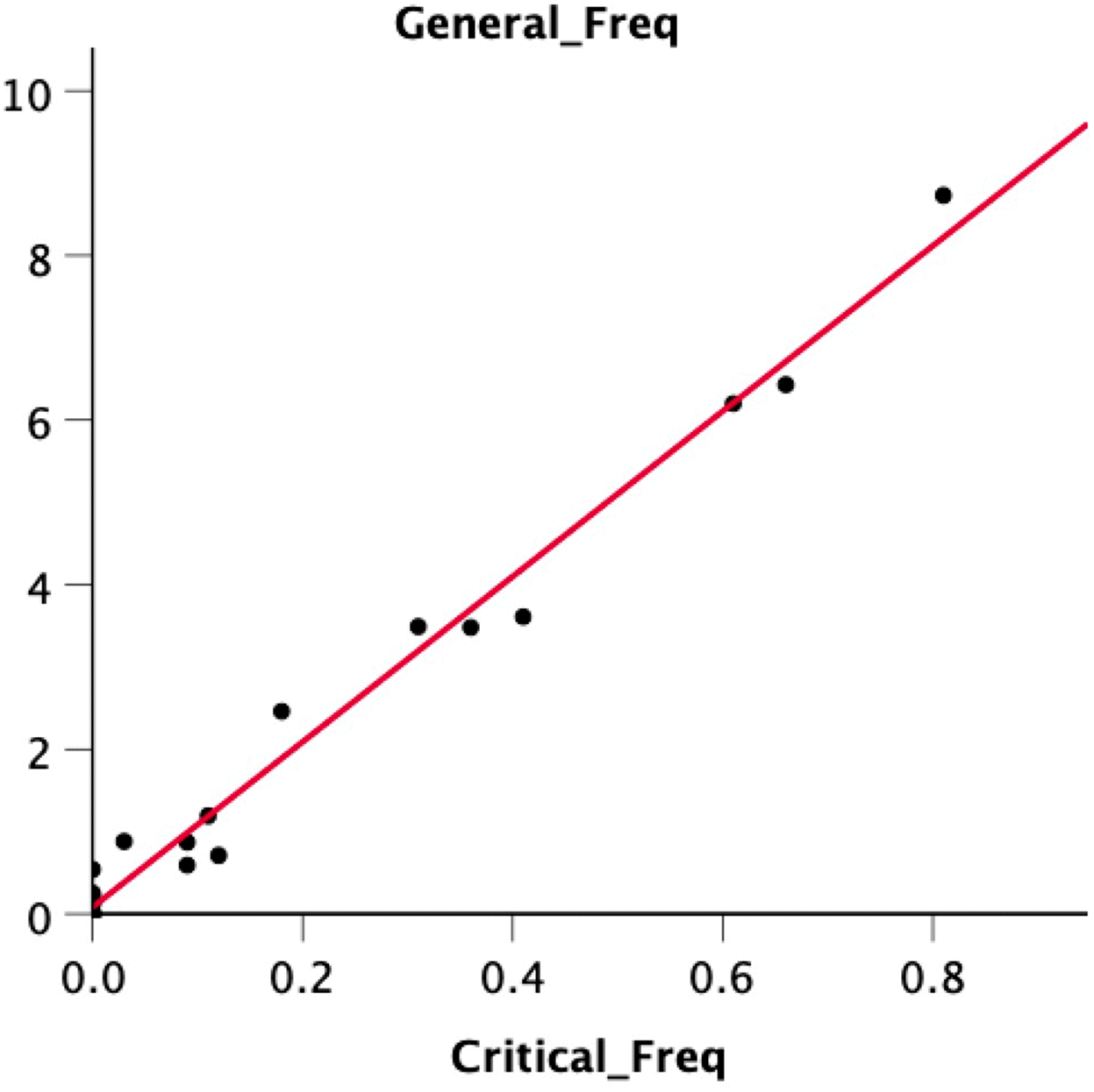
Fitting the relevance of the general frequency and the critical frequency.

From Table 3, it can be drawn that the diachronic thickness of *be going to* lasts for 110 years (1580−1690) and the synchronic intensity of *be going to* amounts to 0.81 per million words. To explore the relevance between the general frequency and the critical frequency, linear regression test is applied. The group of data in the column labelled as “general frequency” in Table 3 are taken as dependent variable while those in the “critical frequency” column are taken as independent variable. The relevance between the general frequency and the critical frequency is shown in the regression plot in Fig 2.

It can be drawn from Fig 2 that the regression model reveals a strong positive linear relationship between *x* and *y*, with the fitted equation y=10.046x+0.08. The determination coefficient *R*^2^ = 0.984 indicates that 98.4% of the variance in *y* is accounted for by x. This high value reflects an excellent model fit and suggests that the two frequency measures are very strongly associated. The slope (10.046) indicates a directly proportional relationship: a one-unit increase in *x* corresponds to an increase of approximately 10.046 units in *y*. The intercept (0.08) estimates the value of *y* when *x* is zero. Specifically, this demonstrates that the critical frequency is linearly related to the general frequency. The general frequency increases with the increase of the critical frequency.

## Discussion

Grammaticalization is closely related to subjectification. According to Traugott [40], subjectification is characteristic of “all domains of grammaticalisation”. Traugott [44] further discussed the degree and step in which subjectification occurs in grammaticalization. Furthermore, Traugott [44] put that subjectification is the subtype of the mechanism of syntactic change. As mentioned in Introduction, the study of the critical frequency can be regarded as the integrating study of frequency and mechanism of grammaticalization. In the previous Section, the critical frequency of *be going to* has been determined. Therefore, the relationship between the grammaticalization of *be going to* and the mechanism of grammaticalization of *be going to* remains to be fully explored. In this section, the relationship between the grammaticalization and the subjectification of *be going to* will be discussed.

Traugott [40] defined subjectification in grammaticalization as, “the development of a grammatically identifiable expression of speaker belief or speaker attitude to what is said.” Traugott [40] demonstrated the dimensions along which subjectification in grammaticalization operates as “objective meaning→subjective meaning; non-epistemic modality→epistemic modality; syntactic subject→speaking subject; full, free form→bonded form.”

Objective meaning→subjective meaning. *Go* originally means a concrete movement in space while future marker *be going to* bears subjective meaning. Hopper & Traugott [25] put that as a future *be going to* is more based in “the speaker’s subjective attitude and perspective” on what is being talked about. In the grammaticalization of *be going to*, some new meanings arise, which are more abstract and “speaker-based” meanings [25]. Diewald [45] directly equated “speaker-based” meaning to “subjective” meaning.

Non-epistemic modality→epistemic modality. Traugott [40] gave the example: “An accident has been reported on Crockett Boulevard. —That *is going to* be South of Crockett.” Traugott [40] held that its association with intention and planned, likely eventhood, suggests that it is also modalised. In this example, *be going to* is roughly equivalent to *would*, which demonstrates strong epistemic modality.

Syntactic subject→speaking subject. Langacker [46] gave two examples:

19 (a) She was going to close the door
(b) An earthquake is going to destroy that town.

Example (a) describes an objective movement along a spatial path by the subject of the sentence (she). And the subject initiates the action of closing the door at the end of the spatial path. Example (b) bears a future coloring in which the subject of the sentence does not move along any spatial path. The speaker (as opposed to the subject) predicts the occurrence of the event. Thus, the conception of imminence or predictability arises.

Full, free form→bonded form. In the last stage of the grammaticalization of *be going to*, it undergoes phonological reduction, which is typical of auxiliary. As there is no longer phrasal boundary between -*ing* and *to*, the three morphemes *go* -*ing to* are reduced to one (*gonna*). Phonological reduction is also a typical feature of more advanced level of grammaticalization.

Traugott [40] and Langacker [46] both considered *be going to* as a typical example of subjectification in grammaticalization.

According to Yang [47], the structure of *be going to V* can be regarded as a hypotactic clause complex composed by a finite clause and a non-finite clause with its structural weight and semantic weight on the finite clause *be going*. With the semantic bleaching of *going*, *be going to* gets fully grammaticalized. The semantic weight of *be going to* shifts to non-finite clause *to V*, and the clause complex shifts to a simple clause. The appearance of rank downgrade thus gives rise to ideational metaphor in the Hallidayan sense [39]. The relationship between grammaticalization and grammatical metaphor is accordingly established, that is, grammaticalization gives rise to ideational metaphor. The grammaticalization process of *be going to* is also the emerging process of grammatical metaphor. The emergence of grammatical metaphor is attributed to the reorganization of meaning in new grammatical structures [48].

Subjectification gives rise to modality metaphor. With the semantic bleaching of the verbal phrase *be going* bearing no modality, the structure of *be going to* grammaticalizes into an auxiliary verb to express the speaker’s attitude and willingness. The subjective modality thus appears. The appearance of modality can be considered as a special kind of modality metaphor. When the speaker’s participation is demonstrated, the reorganized grammatical structure is equipped with interpersonal meaning. Subjectification accordingly appears. The bleaching of subjectified meaning may give rise to grammaticalization. The process of grammaticalization, subjectification and grammatical metaphor all share the characteristic of unidirectional shift [49]. The grammaticalized modal verb *be going to* has the potential to be further bleached into an auxiliary verb. The grammaticalization process of *be going to*, from space to time, to modal verb and ultimately to auxiliary verb, forms a cline.

## For further research

This study serves as a trial exploration for the critical frequency of the construction *be going to* by a corpus-based approach. This study reveals the critical frequency of grammaticalization of *be going to* and confirms the strong correlation between its critical frequency and general frequency. In the future study, the universality and regularity of critical frequency of morphemes and constructions and its relevance with general frequency call for further exploration. The following two topics may be the research focus: (1) the generality of critical frequency (i.e., diachronic thickness and synchronic intensity) across languages; (2) the transcategory (across morphemes and constructions) generality of critical frequency.

## Conclusion

In this study, the grammaticalization process of *be going to* is classified into 6 stages and the contexts for the grammaticalization of *be going to* are categorized into initial stage, critical context and isolating context. Based on the above, 159 verbs appearing in *be going to+ V* are regarded as constituting the critical context of grammaticalization of *be going to*. The critical frequency of *be going to* is confirmed. The diachronic thickness of *be going to* lasts for 110 years (1580–1690) and the synchronic intensity of *be going to* amounts to 0.81 pmw. Furthermore, the relevance of critical frequency and general frequency is explored. There lies high correlation between critical frequency and general frequency of *be going to*.

## Supporting information

S1 File The raw data in Figure 1. (DOCX)

S2 File The raw data in Figure 2. (DOCX)

S3 File The high-frequency words appearing in the constructions of *go and V* and *go to V.* (DOCX)

S4 File Appendix A. (DOCX)

S5 File Appendix B. (DOCX)
