# Supplementary material for: Revisiting the grammaticalization of future be going to: A corpus-based approach
Source: PLoS One. 2026 Jul 24;21(7):e0352674. doi: 10.1371/journal.pone.0352674 (PMC13399480; doi:10.1371/journal.pone.0352674)
Supplement: S1 File — (DOCX) [file pone.0352674.s001.docx]

**Supporting Information Files**

**The raw data in Figure 1**

The raw data in Figure 1 are from Table 1.

The raw data in Table 1 are retrieved from the Google Books Corpus by SQ1:

1. The frequency of the construction *go and V* and *go to V* during the years 1500—1700

| Time | Frequency of the construction *go and V*  (per million words) | Frequency of the construction *go to V*  (per million words) |
| --- | --- | --- |
| 1500 | 0 | 0 |
| 1510 | 0 | 0 |
| 1520 | 0 | 0 |
| 1530 | 0 | 0 |
| 1540 | 0 | 0 |
| 1550 | 0 | 0 |
| 1560 | 0 | 0 |
| 1570 | 0 | 0 |
| 1580 | 2.5 | 0 |
| 1590 | 2 | 22 |
| 1600 | 0 | 0 |
| 1610 | 0 | 0 |
| 1620 | 0 | 30 |
| 1630 | 0 | 0 |
| 1640 | 2.5 | 7.5 |
| 1650 | 5 | 7.5 |
| 1660 | 75 | 128.75 |
| 1670 | 0 | 0 |
| 1680 | 18.75 | 43.75 |
| 1690 | 11 | 28 |
| 1700 | 20.91 | 80.91 |
| Total | 137.66 | 348.41 |

1. The frequency of the construction *go and V* and *go to V* during the years 1710—2000

| Time | Frequency of the construction *go and V*  (per million words) | Frequency of the construction *go to V*  (per million words) |
| --- | --- | --- |
| 1710 | 15 | 42.5 |
| 1720 | 16.67 | 45 |
| 1730 | 14.67 | 14 |
| 1740 | 8.33 | 18.89 |
| 1750 | 9.23 | 27.69 |
| 1760 | 21.05 | 123.68 |
| 1770 | 4.35 | 17.83 |
| 1780 | 10 | 34.79 |
| 1790 | 11.84 | 31.03 |
| 1800 | 12.05 | 32.22 |
| 1810 | 11.14 | 30.64 |
| 1820 | 12.38 | 30.60 |
| 1830 | 13.4 | 33.98 |
| 1840 | 15.07 | 35.14 |
| 1850 | 15.55 | 39.17 |
| 1860 | 17.11 | 48.27 |
| 1870 | 17.04 | 50.42 |
| 1880 | 15.78 | 50.35 |
| 1890 | 19.24 | 65.65 |
| 1900 | 17.39 | 70.16 |
| 1910 | 14.24 | 79.49 |
| 1920 | 14.43 | 86.13 |
| 1930 | 14.03 | 92.5 |
| 1940 | 11.21 | 98.05 |
| 1950 | 10.87 | 89.31 |
| 1960 | 9.35 | 80.52 |
| 1970 | 7.96 | 87.06 |
| 1980 | 6.91 | 99.63 |
| 1990 | 7.49 | 120.58 |
| 2000 | 7.91 | 151.37 |
| total | 381.69 | 1826.65 |
