# Supplementary material for: Revisiting the grammaticalization of future be going to: A corpus-based approach
Source: PLoS One. 2026 Jul 24;21(7):e0352674. doi: 10.1371/journal.pone.0352674 (PMC13399480; doi:10.1371/journal.pone.0352674)
Supplement: S2 File — (DOCX) [file pone.0352674.s002.docx]

**Supporting Information Files**

**The raw data in Figure 2**

The raw data in Figure 2 are from Table 3. The critical frequency of *be going to* is obtained by the sum of the frequency of 159 verbs in *be going to V* construction in EEBO corpus. The 159 verbs are listed in the appendix respectively.
