# Supplementary material for: Revisiting the grammaticalization of future be going to: A corpus-based approach
Source: PLoS One. 2026 Jul 24;21(7):e0352674. doi: 10.1371/journal.pone.0352674 (PMC13399480; doi:10.1371/journal.pone.0352674)
Supplement: S3 File — (DOCX) [file pone.0352674.s003.docx]

**Supporting Information Files**

**The high-frequency words appearing in the constructions of *go and V* and *go to V***

(1) The high-frequency words appearing in the construction of *go and V* are retrieved by SQ1 in EEBO. The top 50, 100 and 200 high-frequency words are listed below.

The top 200 high-frequency words in construction *go and V*

|  | word | frequency |  | word | frequency |
| --- | --- | --- | --- | --- | --- |
| 1 | come | 791 | 101 | inform | 33 |
| 2 | tell | 609 | 102 | cut | 33 |
| 3 | take | 520 | 103 | work | 33 |
| 4 | preach | 490 | 104 | comfort | 32 |
| 5 | see | 476 | 105 | charge | 32 |
| 6 | make | 397 | 106 | ride | 32 |
| 7 | return | 374 | 107 | relieve | 31 |
| 8 | be | 319 | 108 | draw | 31 |
| 9 | com | 292 | 109 | flee | 30 |
| 10 | seek | 283 | 110 | camp | 30 |
| 11 | do | 262 | 111 | bid | 30 |
| 12 | sell | 247 | 112 | seize | 30 |
| 13 | meet | 227 | 113 | pay | 30 |
| 14 | serve | 216 | 114 | prosper | 29 |
| 15 | visit | 215 | 115 | eat | 29 |
| 16 | fetch | 201 | 116 | demand | 29 |
| 17 | sit | 193 | 117 | yield | 29 |
| 18 | teach | 193 | 118 | talk | 28 |
| 19 | dwell | 167 | 119 | kneel | 28 |
| 20 | find | 163 | 120 | knock | 27 |
| 21 | hang | 151 | 121 | plant | 27 |
| 22 | say | 147 | 122 | stay | 27 |
| 23 | bring | 145 | 123 | dig | 27 |
| 24 | besiege | 145 | 124 | follow | 27 |
| 25 | fight | 142 | 125 | confess | 27 |
| 26 | give | 141 | 126 | beg | 27 |
| 27 | pray | 141 | 127 | assault | 27 |
| 28 | show | 135 | 128 | throw | 27 |
| 29 | speak | 133 | 129 | touch | 27 |
| 30 | lie | 125 | 130 | turn | 26 |
| 31 | prepare | 121 | 131 | arm | 26 |
| 32 | hide | 117 | 132 | attend | 25 |
| 33 | encamp | 115 | 133 | encounter | 25 |
| 34 | leave | 113 | 134 | perform | 25 |
| 35 | live | 110 | 135 | place | 24 |
| 36 | lodge | 110 | 136 | rest | 24 |
| 37 | gather | 109 | 137 | trade | 24 |
| 38 | set | 105 | 138 | raise | 23 |
| 39 | lay | 99 | 139 | send | 23 |
| 40 | buy | 99 | 140 | possess | 23 |
| 41 | receive | 97 | 141 | let | 23 |
| 42 | call | 89 | 142 | attack | 23 |
| 43 | put | 86 | 143 | bear | 23 |
| 44 | stand | 84 | 144 | disciple | 22 |
| 45 | join | 83 | 145 | seke | 22 |
| 46 | sin | 80 | 146 | burn | 21 |
| 47 | offer | 75 | 147 | will | 21 |
| 48 | hear | 75 | 148 | weep | 20 |
| 49 | present | 74 | 149 | break | 20 |
| 50 | look | 74 | 150 | command | 20 |
| 51 | search | 74 | 151 | beseege | 20 |
| 52 | acquaint | 73 | 152 | destroy | 20 |
| 53 | fall | 71 | 153 | report | 20 |
| 54 | wash | 71 | 154 | inhabit | 20 |
| 55 | se | 70 | 155 | provide | 20 |
| 56 | get | 69 | 156 | sleep | 19 |
| 57 | cry | 69 | 157 | spoil | 19 |
| 58 | bury | 69 | 158 | desire | 19 |
| 59 | carry | 69 | 159 | enjoy | 19 |
| 60 | cast | 67 | 160 | accuse | 19 |
| 61 | ask | 66 | 161 | confer | 19 |
| 62 | pass | 66 | 162 | vanish | 19 |
| 63 | walk | 60 | 163 | fill | 18 |
| 64 | deliver | 58 | 164 | submit | 18 |
| 65 | discover | 58 | 165 | refresh | 18 |
| 66 | learn | 55 | 166 | mete | 18 |
| 67 | kiss | 55 | 167 | ioyne | 17 |
| 68 | view | 55 | 168 | proclaim | 17 |
| 69 | open | 54 | 169 | repair | 17 |
| 70 | try | 52 | 170 | dispatch | 17 |
| 71 | spend | 51 | 171 | din | 17 |
| 72 | depart | 51 | 172 | advise | 17 |
| 73 | consult | 49 | 173 | advertise | 16 |
| 74 | wait | 49 | 174 | commeth | 16 |
| 75 | complain | 47 | 175 | choose | 16 |
| 76 | embrace | 46 | 176 | defend | 16 |
| 77 | die | 45 | 177 | entertain | 16 |
| 78 | assail | 45 | 178 | examine | 16 |
| 79 | salute | 45 | 179 | remain | 16 |
| 80 | kill | 45 | 180 | pull | 16 |
| 81 | enquire | 43 | 181 | surprise | 16 |
| 82 | sacrifice | 42 | 182 | travel | 15 |
| 83 | smite | 40 | 183 | treat | 15 |
| 84 | keep | 40 | 184 | write | 15 |
| 85 | enter | 40 | 185 | invite | 15 |
| 86 | have | 40 | 186 | marry | 15 |
| 87 | baptize | 38 | 187 | read | 15 |
| 88 | humble | 38 | 188 | secure | 15 |
| 89 | succour | 38 | 189 | assist | 15 |
| 90 | run | 37 | 190 | consider | 14 |
| 91 | build | 37 | 191 | feed | 14 |
| 92 | declare | 37 | 192 | hir | 14 |
| 93 | lose | 36 | 193 | lead | 14 |
| 94 | worship | 36 | 194 | know | 14 |
| 95 | drink | 35 | 195 | land | 14 |
| 96 | conquer | 35 | 196 | plead | 14 |
| 97 | help | 34 | 197 | sup | 14 |
| 98 | play | 34 | 198 | murder | 13 |
| 99 | spake | 34 | 199 | observe | 13 |
| 100 | settle | 33 | 200 | post | 13 |

(2) The high-frequency words appearing in the construction of *go to V* are retrieved by SQ1 in EEBO. The top 50, 100 and 200 high-frequency words are listed below.

The top 200 high-frequency words in construction *go to V*

|  | word | frequency |  | word | frequency |
| --- | --- | --- | --- | --- | --- |
| 1 | see | 1391 | 101 | assist | 41 |
| 2 | visit | 1115 | 102 | draw | 40 |
| 3 | meet | 1038 | 103 | demand | 40 |
| 4 | seek | 841 | 104 | assail | 38 |
| 5 | take | 726 | 105 | attend | 38 |
| 6 | be | 662 | 106 | aid | 38 |
| 7 | make | 625 | 107 | treat | 38 |
| 8 | wrack | 575 | 108 | suffer | 37 |
| 9 | rest | 472 | 109 | provide | 37 |
| 10 | fight | 381 | 110 | build | 36 |
| 11 | prepare | 373 | 111 | kill | 35 |
| 12 | hear | 346 | 112 | sleep | 35 |
| 13 | find | 331 | 113 | study | 34 |
| 14 | besiege | 299 | 114 | read | 33 |
| 15 | fetch | 296 | 115 | counsel | 33 |
| 16 | ruin | 247 | 116 | fish | 33 |
| 17 | give | 242 | 117 | feed | 32 |
| 18 | view | 222 | 118 | destroy | 32 |
| 19 | receive | 220 | 119 | refresh | 32 |
| 20 | do | 210 | 120 | raise | 31 |
| 21 | wait | 200 | 121 | inquire | 31 |
| 22 | possess | 194 | 122 | beseege | 31 |
| 23 | battle | 171 | 123 | cast | 30 |
| 24 | serve | 166 | 124 | deliver | 30 |
| 25 | pray | 155 | 125 | comfort | 29 |
| 26 | dwell | 154 | 126 | save | 29 |
| 27 | preach | 148 | 127 | say | 29 |
| 28 | lodge | 146 | 128 | spoil | 28 |
| 29 | have | 139 | 129 | write | 28 |
| 30 | buy | 136 | 130 | bury | 28 |
| 31 | decay | 129 | 131 | advertise | 28 |
| 32 | se | 129 | 132 | go | 28 |
| 33 | field | 125 | 133 | behold | 27 |
| 34 | look | 124 | 134 | burn | 27 |
| 35 | live | 123 | 135 | rob | 27 |
| 36 | plough | 120 | 136 | shore | 27 |
| 37 | speak | 119 | 137 | surprise | 26 |
| 38 | mete | 116 | 138 | bathe | 26 |
| 39 | prove | 112 | 139 | entertain | 26 |
| 40 | discover | 112 | 140 | ioyne | 26 |
| 41 | tell | 105 | 141 | mount | 26 |
| 42 | encounter | 99 | 142 | rack | 25 |
| 43 | walk | 96 | 143 | labour | 25 |
| 44 | play | 95 | 144 | accompany | 25 |
| 45 | gather | 95 | 145 | settle | 25 |
| 46 | call | 92 | 146 | service | 25 |
| 47 | search | 91 | 147 | work | 25 |
| 48 | pay | 90 | 148 | answer | 24 |
| 49 | salute | 89 | 149 | bid | 24 |
| 50 | drink | 84 | 150 | complain | 24 |
| 51 | put | 83 | 151 | cut | 24 |
| 52 | land | 82 | 152 | repose | 24 |
| 53 | seke | 82 | 153 | open | 23 |
| 54 | join | 79 | 154 | let | 23 |
| 55 | lay | 78 | 155 | guard | 23 |
| 56 | help | 78 | 156 | confer | 23 |
| 57 | kiss | 77 | 157 | breakfast | 23 |
| 58 | try | 76 | 158 | wrake | 23 |
| 59 | sacrifice | 73 | 159 | seize | 22 |
| 60 | lie | 73 | 160 | catch | 22 |
| 61 | get | 71 | 161 | marry | 22 |
| 62 | hunt | 70 | 162 | pull | 22 |
| 63 | encamp | 70 | 163 | recreate | 21 |
| 64 | die | 70 | 164 | forage | 21 |
| 65 | conquer | 68 | 165 | break | 21 |
| 66 | wreck | 68 | 166 | rut | 21 |
| 67 | ask | 67 | 167 | spend | 21 |
| 68 | charge | 64 | 168 | sell | 20 |
| 69 | offer | 64 | 169 | win | 20 |
| 70 | worship | 63 | 170 | beg | 20 |
| 71 | succour | 63 | 171 | examine | 20 |
| 72 | show | 62 | 172 | govern | 20 |
| 73 | travel | 62 | 173 | return | 20 |
| 74 | pot | 61 | 174 | punish | 20 |
| 75 | enquire | 61 | 175 | render | 19 |
| 76 | eat | 61 | 176 | execute | 19 |
| 77 | set | 60 | 177 | subdue | 19 |
| 78 | consult | 59 | 178 | sup | 19 |
| 79 | carry | 59 | 179 | shrift | 18 |
| 80 | relieve | 58 | 180 | satisfy | 18 |
| 81 | bring | 56 | 181 | waste | 18 |
| 82 | learn | 54 | 182 | dust | 18 |
| 83 | hide | 53 | 183 | declare | 18 |
| 84 | acquaint | 53 | 184 | attack | 18 |
| 85 | assault | 52 | 185 | celebrate | 18 |
| 86 | dine | 52 | 186 | conduct | 18 |
| 87 | recover | 51 | 187 | choose | 18 |
| 88 | present | 49 | 188 | plant | 18 |
| 89 | market | 48 | 189 | hold | 17 |
| 90 | defend | 48 | 190 | bear | 17 |
| 91 | keep | 47 | 191 | anoint | 17 |
| 92 | ship | 47 | 192 | crave | 17 |
| 93 | inhabit | 45 | 193 | cure | 17 |
| 94 | perform | 45 | 194 | ease | 17 |
| 95 | know | 43 | 195 | embark | 17 |
| 96 | enjoy | 43 | 196 | turn | 17 |
| 97 | embrace | 43 | 197 | spy | 17 |
| 98 | sit | 43 | 198 | stay | 16 |
| 99 | wash | 43 | 199 | talk | 16 |
| 100 | pass | 42 | 200 | send | 16 |
