# Supplementary material for: Revisiting the grammaticalization of future be going to: A corpus-based approach
Source: PLoS One. 2026 Jul 24;21(7):e0352674. doi: 10.1371/journal.pone.0352674 (PMC13399480; doi:10.1371/journal.pone.0352674)
Supplement: S4 File — (DOCX) [file pone.0352674.s004.docx]

**Supporting Information Files**

**Appendix A**

28 words cooccurring from both the 50 high-frequency words in “[go] to [v*]” and in “[go] and [v*]”: SEE, TELL, VISIT, TAKE, MEET, PREACH, SEEK, MAKE, BE, FIGHT, DO, PREPARE, HEAR, FIND, SERVE, BESIEGE, FETCH, GIVE, DWELL, RECEIVE, PRAY, LODGE, SPEAK, BUY, LOOK, LIVE, GATHER, CALL.

68 words cooccurring from both the 100 high-frequency words in “[go] to [v*]” and in “[go] and [v*]”: SEE, TELL, VISIT, TAKE, MEET, PREACH, SEEK, MAKE, BE, FIGHT, DO, PREPARE, HEAR, FIND, SERVE, BESIEGE, FETCH, SIT, GIVE, VIEW, DWELL, RECEIVE, WAIT, BRING, PRAY, SHOW, LODGE, SPEAK, HAVE, LIE, BUY, HIDE, SE, ENCAMP, LOOK, LIVE, GATHER, SET, LAY, DISCOVER, CALL, PUT, WALK, PLAY, JOIN, OFFER, SEARCH, PRESENT, SALUTE, DRINK, ACQUAINT, WASH, GET, HELP, KISS, TRY, CARRY, SACRIFICE, ASK, PASS, DIE, CONQUER, LEARN, WORSHIP, SUCCOUR, CONSULT, ENQUIRE, EMBRACE, KEEP.

141 words cooccurring from both the 200 high-frequency words in “[go] to [v*]” and in “[go] and [v*]”: SEE, TELL, VISIT, TAKE, MEET, PREACH, SEEK, MAKE, BE, RETURN, REST, FIGHT, DO, PREPARE, SELL, HEAR, FIND, SERVE, BESIEGE, FETCH, SIT, GIVE, VIEW, DWELL, RECEIVE, WAIT, SAY, POSSESS, BRING, PRAY, SHOW, LODGE, SPEAK, HAVE, LIE, BUY, HIDE, SE, ENCAMP, LOOK, LIVE, GATHER, SET, METE, LAY, DISCOVER, CALL, ENCOUNTER, PUT, WALK, PLAY, JOIN, OFFER, SEARCH, PAY, PRESENT, SALUTE, DRINK, ACQUAINT, LAND, SEKE, WASH, GET, HELP, KISS, BURY, TRY, CARRY, SACRIFICE, CAST, ASK, PASS, DELIVER, DIE, CONQUER, LEARN, CHARGE, OPEN, WORSHIP, SPEND, SUCCOUR, CONSULT, TRAVEL, COMPLAIN, ENQUIRE, EMBRACE, EAT, ASSAIL, KILL, RELIEVE, KEEP, ASSAULT, DEFEND, BUILD, DECLARE, INHABIT, PERFORM, KNOW, ENJOY, SETTLE, ASSIST, CUT, DRAW, WORK, DEMAND, COMFORT, ATTEND, TREAT, PROVIDE, BID, SEIZE, SLEEP, READ, FEED, TALK, DESTROY, REFRESH, RAISE, PLANT, STAY, BESEEGE, BEG, SPOIL, WRITE, TURN, ADVERTISE, BURN, SURPRISE, SEND, ENTERTAIN, IOYNE, LET, ATTACK, BEAR, BREAK, CONFER, MARRY, PULL, EXAMINE, CHOOSE, SUP.
