# Supplementary material for: Revisiting the grammaticalization of future be going to: A corpus-based approach
Source: PLoS One. 2026 Jul 24;21(7):e0352674. doi: 10.1371/journal.pone.0352674 (PMC13399480; doi:10.1371/journal.pone.0352674)
Supplement: S5 File — (DOCX) [file pone.0352674.s005.docx]

**Supporting Information Files**

**Appendix B**

159 words occurring in “be going to + V”, which will constitute the critical contexts for the grammaticalization of *be going to*:

reply, describe, partake, mention, add, employ, battle, name, march, prescribe, recount, produce, quit, reveal, spring, tempt, cite, condemn, hit, hammer, imitate, express, expire, enlarge, decay, alter, demonstrate, convey, cleave, unlade, wrestle, repass, quote, re-enter, recite, oblige, overflow, mislead, moore, object, level, melt, mask, poison, pin, pavvn, precipitate, redouble, rehearse, recreut, reassume, rebel, push, re-count, recount, rain, retrench, roost, reverse, reward, satiate, scale, reprint, vindicate, vex, vanquish, vittell, vset, wage, waint, weigh’t, wipe, withdraw, travel, tipple, transcribe, unbuckle, strive, subjoin, subjoin, start, steer, step, sting, tak, swallow, swap, themperour, tender, tear, shower, shrift, speap, skail, slip, solemnize, sound, separate, shoe, clean, cofss, conceive, complete, cashier, cheapen, crush, contemplate, contract, delineate, deduce, bark, battell, banter, bend, beget, bite, bleed, boil, besige, bestride, buckle, account, advance, annex, assign, divine, dilute, direct, detach, dissect, distil, distribute, dive, displease, enumerate, exalt, ensnare, erect, exceed, exchange, expand, expiate, extirpate, field, hadrianople, forbid, forge, impute, infest, inflict, limbark, kick, lash, horseback, huddle, involve, ingag, initiate, ink.
