# Supplementary material for: Revisiting the grammaticalization of future be going to: A corpus-based approach
Source: PLoS One. 2026 Jul 24;21(7):e0352674. doi: 10.1371/journal.pone.0352674 (PMC13399480; doi:10.1371/journal.pone.0352674)
Supplement: Attachment — Submitted filename: Response to Reviewers.docx [file pone.0352674.s007.docx]

Rebuttal Letter

We sincerely appreciate the editor and reviewers for their thoughtful feedback and constructive suggestions, which have greatly helped improve our manuscript. The following are one-to-one responses to the comments and suggestions.

Reviewer Comments

Reviewer 1

1. Comment: Definition of Critical Context and Ambiguity. “Providing specific examples of sentences where ‘be going to’ initially cause ambiguity in the critical context would be beneficial”.

Response: In the six stages of the grammaticalization of future *be going to*, the author gives more examples to explain the grammaticalization in detail, especially how ambiguity arises in the critical context. The revised paper reads as “In Example (7), *be going to* is followed by an action verb *kill*, and *be going to* here has two interpretations. The first one is ‘I am moving from one place to another with the aim of killing the president’ while the second one is ‘I will kill the president in the future’. Thus, ambiguous interpretations arise, which forms the critical context for the grammaticalization of *be going to*.” In the critical context, the pragmatic inference can be fulfilled. *be going to*, originally meaning movement in space, can refer to future in time by means of metaphor. The critical context provides room to trigger grammaticalization.

2. Comment: Discussion on “go” Winning the Competition. “a deeper discussion on why ‘go’ had a higher frequency compared to ‘come’ and ‘leave’ in contexts leading to grammaticalization would strengthen the argument”.

Response: The three verbs of *go*, *come* and *leave* are unmarked for the manner of movement, that is, they do not specify the detail of movement. All of them have the potential to grammaticalize into future markers. In the revision, the reason why *come* and *leave* are not grammaticalized are explained. As for *leave*, “the frequency of *leave* is too low to be further grammaticalized.” As for *come*, “out of the top 10 high-frequency words, 5 coincide with those from SQ2. They are *take* (with its frequency of 23), *see* (20), *meet* (15), *be* (14) and *make* (12). The fact means that there does exist critical context for the potential grammaticalization of *come* into a future marker. But the relative low frequency has not yet reached the threshold for the grammaticalization of *come*.” In conclusion, the high critical frequency of *be going to* leads to the repeated pragmatic inference, which triggers grammaticalization. The frequency of *be leaving to* is too low to trigger grammaticalization. The critical frequency of *be coming to* is not high enough to trigger grammaticalization.

3. Comment: Integration of Criteria. “It would be helpful to explicitly illustrate how these verbs, or similar examples, demonstrate the ‘isolating context’, linking this back to the categorization of verbs for data retrieval.”

Response: In this paper, we categorize three types of contexts for the investigation of the grammaticalization process of future *be going to*: initial stage, critical context and isolating context. The demonstration of the three types of contexts can be seen in Line 162-166. In the initial stage, grammaticalization is not triggered. *be going to* only means movement in space, so it is followed by action verbs. Only in the critical context, ambiguity arises. That is to say *be going to* can refer to either movement in space or future in time. As mentioned in Line 91, “the isolating context is incompatible with some salient properties of the source meaning”. In the isolating context, *be going to* only refers to future in time in all contexts. *Be going to* can be followed by verbs of mental experience or stative verbs.

Correspondingly, verbs can be classified into three categories. (1) verbs in initial stage: these verbs will not cause ambiguity; (2) verbs in critical context: these verbs will cause ambiguity; (3) verbs in isolating context: mental verbs or stative verbs. How to get these three categories of verbs is demonstrated in Line 358-368 of the revised paper.

4. Comment: Future Exploration. “outlining specific avenues for such future exploration could demonstrate a clearer path forward for this line of research.”

Response: In the revision, Section “for Further Research” is added to give the two topics in future research. The generality of critical frequency will be the focus of future exploration. It includes the following two topics. The first one is the generality of critical frequency across languages; the second one is the transcategory (across morphemes and constructions) generality of critical frequency.

Reviewer 2

1. Comment: Technical Soundness and Data-Conclusion Alignment. “The key construct—critical frequency—is not defined with adequate statistical or methodological precision.”

Response: The revision incorporates the research frontier, such as Peng (2011) and Peng (2020) to demonstrate the validity of critical frequency. Peng (2011) investigated the critical frequency of some morphemes and constructions in Chinese. By determining the critical frequency as well as the general frequency of these morphemes and constructions, Peng (2011) proved the validity of critical frequency in grammaticalization. Peng (2020) further explored two issues about critical frequency: (1) whether there exists positive correlation relationship between general frequency and critical frequency; (2) what is the minimum of critical frequency to conventionalize pragmatic implicature? All these works prove the validity of critical frequency theory.

2. Comment: Conceptual and Theoretical Clarity. “The six-stage model of grammaticalization is useful but under-supported by empirical evidence beyond surface frequency patterns.”

Response: In the revision, the six stages of grammaticalization of future *be going to* are expounded in detail respectively. In Line 153-169, the morphosyntactic characteristics of the six stages are demonstrated. Especially in the fifth stage, the initial stage, the critical context and the isolating context are further manifested. In Line 170-184, examples from the corpus are given to each stage. In Line 185-218, each example is explained in detail. By doing so, the six stages of grammaticalization of future *be going to* are demonstrated by empirical evidence.

3. Comment: Conceptual and Theoretical Clarity. The definition of “synchronic intensity” and “diachronic thickness” is not universally standardized and needs more careful operationalization and justification.

Response: As in 1, the revision incorporates the research frontier, such as Peng (2011) and Peng (2020) to demonstrate the validity of synchronic intensity and diachronic thickness. Peng (2011) investigated the synchronic intensity and diachronic thickness of some morphemes and constructions in Chinese. The former includes “*shi* (时)” while the latter covers the competition between two non-structural strings of “yin (因)+er (而)” and “yin (因)+yi (以)”. Moreover, Peng (2020) further elaborated on two issues about critical frequency (i.e., synchronic intensity and diachronic thickness): (1) whether there exists positive correlation relationship between general frequency and critical frequency; (2) what is the minimum of critical frequency to conventionalize pragmatic implicature? All the works prove the validity of synchronic intensity and diachronic thickness. All the above can be seen in Line 116-125 in the revision.

4. Comment: The discussion on subjectification and grammatical metaphor, although relevant, appears as an add-on rather than being fully integrated with the data analysis.

Response: In the revision, subjectification in grammaticalization is rigorously demonstrated. Firstly, the dimensions along which subjectification in grammaticalization operates raised in Traugott (1995) is introduced. Then, how the diachronic change of *be going to* meets those dimensions is demonstrated respectively. Therefore, the diachronic change of *be going to* belongs to subjectification in grammaticalization. Then the relationship between subjectification, grammaticalization and grammatical metaphor is accordingly discussed. All the above can be seen in Line 385-414 in the revision.

5. Comment: Language and Presentation.

Response: The revision has been thoroughly modified by the author and professional academic editors to guarantee its idiomaticity, which will meet the standard of PLOS ONE.

6. Comment: Statistical Analysis. Reviewer 2 raises the following questions: (1) No confidence intervals, standard errors, or effect sizes are reported; (2) Assumptions behind the regression model are not discussed; (3) any interrater reliability or objective metric is not used; (4) The lack of inferential statistical thresholds for defining “critical frequency”.

Response: As to (2), in the paper chi-square test is applied in Figure 1. Linear regression is applied to fit the data in Figure 2 and the data are homogeneously distributed. Although (1), (3) and (4) are illuminating, the present paper is to determine the relationship between the critical frequency and the general frequency and only linear regression test is used, which is only related to (2).

Note: Places that have been revised are highlighted in blue in the revision.
