# Supplementary material for: Revisiting the grammaticalization of future be going to: A corpus-based approach
Source: PLoS One. 2026 Jul 24;21(7):e0352674. doi: 10.1371/journal.pone.0352674 (PMC13399480; doi:10.1371/journal.pone.0352674)
Supplement: Attachment — Submitted filename: Response_to_Reviewers_auresp_2.docx [file pone.0352674.s008.docx]

Rebuttal Letter

We sincerely appreciate the editor and reviewers for their thoughtful feedback and constructive suggestions, which have greatly helped improve our manuscript. The following are one-to-one responses to the comments and suggestions.

Reviewers Comments

Reviewer 1

1. Comment: Definition of Critical Context and Ambiguity. “Providing specific examples of sentences where ‘be going to’ initially cause ambiguity in the critical context would be beneficial”.

Response: In the six stages of the grammaticalization of future *be going to*, the author gives more examples to explain the grammaticalization in detail, especially how ambiguity arises in the critical context. The revised paper reads as “In Example (7), *be going to* is followed by an action verb *kill*, and *be going to* here has two interpretations. The first one is ‘I am moving from one place to another with the aim of killing the president’ while the second one is ‘I will kill the president in the future’. Thus, ambiguous interpretations arise, which forms the critical context for the grammaticalization of *be going to*.” In the critical context, the pragmatic inference can be fulfilled. *be going to*, originally meaning movement in space, can refer to future in time by means of metaphor. The critical context provides room to trigger grammaticalization.

2. Comment: Discussion on “go” Winning the Competition. “a deeper discussion on why ‘go’ had a higher frequency compared to ‘come’ and ‘leave’ in contexts leading to grammaticalization would strengthen the argument”.

Response: The three verbs of *go*, *come* and *leave* are unmarked for the manner of movement, that is, they do not specify the detail of movement. All of them have the potential to grammaticalize into future markers. In the revision, the reason why *come* and *leave* are not grammaticalized are explained. As for *leave*, “the frequency of *leave* is too low to be further grammaticalized.” As for *come*, “out of the top 10 high-frequency words, 5 coincide with those from SQ2. They are *take* (with its frequency of 23), *see* (20), *meet* (15), *be* (14) and *make* (12). The fact means that there does exist critical context for the potential grammaticalization of *come* into a future marker. But the relative low frequency has not yet reached the threshold for the grammaticalization of *come*.” In conclusion, the high critical frequency of *be going to* leads to the repeated pragmatic inference, which triggers grammaticalization. The frequency of *be leaving to* is too low to trigger grammaticalization. The critical frequency of *be coming to* is not high enough to trigger grammaticalization.

3. Comment: Integration of Criteria. “It would be helpful to explicitly illustrate how these verbs, or similar examples, demonstrate the ‘isolating context’, linking this back to the categorization of verbs for data retrieval.”

Response: In this paper, we categorize three types of contexts for the investigation of the grammaticalization process of future *be going to*: initial stage, critical context and isolating context. The demonstration of the three types of contexts can be seen in Line 177-181. In the initial stage, grammaticalization is not triggered. *be going to* only means movement in space, so it is followed by action verbs. Only in the critical context, ambiguity arises. That is to say *be going to* can refer to either movement in space or future in time. As mentioned in Line 117, “the isolating context is incompatible with some salient properties of the source meaning”. In the isolating context, *be going to* only refers to future in time in all contexts. *Be going to* can be followed by verbs of mental experience or stative verbs.

Correspondingly, verbs can be classified into three categories. (1) verbs in initial stage: these verbs will not cause ambiguity; (2) verbs in critical context: these verbs will cause ambiguity; (3) verbs in isolating context: mental verbs or stative verbs. How to get these three categories of verbs is demonstrated in Line 382-392 of the revised paper.

4. Comment: Future Exploration. “outlining specific avenues for such future exploration could demonstrate a clearer path forward for this line of research.”

Response: In the revision, Section “for Further Research” is added to give the two topics in future research. The generality of critical frequency will be the focus of future exploration. It includes the following two topics. The first one is the generality of critical frequency across languages; the second one is the transcategory (across morphemes and constructions) generality of critical frequency.

Reviewer 2

1. Comment: Technical Soundness and Data-Conclusion Alignment. “The key construct—critical frequency—is not defined with adequate statistical or methodological precision.”

Response:

(1) The frequency in the critical context is termed as the critical frequency. Pragmatic inference can only be fulfilled by the critical frequency. In the paper, how to get the critical frequency of the grammaticalization of *be going to* has been rigorously demonstrated. Moreover, the definition of diachronic thickness and synchronic intensity is presented in Note ⅰ and Note ⅱ respectively.

(2) The revision incorporates the research frontier, such as Peng (2011) and Peng (2020) to demonstrate the validity of critical frequency. Peng (2011) investigated the critical frequency of some morphemes and constructions in Chinese. By determining the critical frequency as well as the general frequency of these morphemes and constructions, Peng (2011) conducted a preliminary exploration of the critical frequency in grammaticalization. Peng (2020) further explored two issues about critical frequency: (1) whether there exists positive correlation relationship between general frequency and critical frequency; (2) what is the minimum of critical frequency to conventionalize pragmatic implicature? These studies have explored the validity of the critical frequency.

2. Comment: Statistical Analysis. Several shortcomings weaken the statistical rigor: (1) No confidence intervals, standard errors, or effect sizes are reported. (2) Assumptions behind the regression model are not discussed. (3) The classification of verbs into “critical” and “non-critical” contexts is not empirically tested, nor is any interrater reliability or objective metric used. (4) The lack of inferential statistical thresholds for defining “critical frequency” raises concerns about replicability.

Response: (1) This quantitative study is based on large, balanced and authoritative diachronic corpora. The sizes of each corpus have been presented in the paper. The study carried out exhaustive retrieval of eligible data through search queries rather than random sampling and both the retrieved data and the results of the study have replicability. All the retrieved data are presented as standardized frequencies per million words to guarantee the comparability of the data across texts of different sizes or from different historical periods.

(2) We have now conducted a comprehensive diagnostic check of the model residuals. This analysis included: a Q-Q plot to assess the normality of the residuals, a residuals-versus-fitted values plot to assess homoscedasticity and linearity. The diagnostic plots confirm that there are no substantial violations of the model assumptions. The residuals closely follow the normal distribution in the Q-Q plot and are distributed homoscedastically around zero in the residual-vs-fitted plot. We would also like to respectfully highlight the specific context of our model, which provides additional, powerful evidence for its validity. The regression equation (y = 10.046x + 0.08) yields an exceptionally high coefficient of determination (R² = 0.984). This indicates that the linear model explains 98.4% of the variance in the data. In such a case of a near-perfect linear fit, the relationship is overwhelmingly deterministic. The model is exceptionally well-specified, and the parameter estimates are highly robust, meaning that the core findings concerning the slope and intercept are fundamentally sound and reliable.

(3) With ambiguity as its distinctive features, critical context of the grammaticalization of *be going to* is rigorously defined (how to get the 159 words) and the critical frequency is determined through logical reasoning. The process remains independent of any interrater. Both the formalization of the critical context and the formulation of search queries are consistent, irrespective of the researcher involved. All data and results are fully replicable.

(4) The critical frequencies of different morphemes and constructions may vary. It can be any value as long as it can trigger the grammaticalization of the grammaticalizing items. For *be going to*, however, we objectively present its critical frequency by formalizing the critical context through logical reasoning and an exhaustive corpus retrieval. This methodology guarantees that the identified critical frequency for the grammaticalization of *be going to* is fully replicable.

3. Comment: Language and Presentation.

Response: The revision has been thoroughly modified by the author and professional academic editors to guarantee its idiomaticity, which will meet the standard of PLOS ONE.

4. Comment: Conceptual and Theoretical Clarity. “The six-stage model of grammaticalization is useful but under-supported by empirical evidence beyond surface frequency patterns.”

Response: In the revision, the six stages of grammaticalization of future *be going to* are expounded in detail respectively. In Line 168-184, the morphosyntactic characteristics of the six stages are demonstrated. Especially in the fifth stage, the initial stage, the critical context and the isolating context are further manifested. In Line 185-199, examples from the corpus are given to each stage. In Line 200-233, each example is explained in detail. By doing so, the six stages of grammaticalization of future *be going to* are demonstrated by empirical evidence.

5. Comment: Conceptual and Theoretical Clarity. The definition of “synchronic intensity” and “diachronic thickness” is not universally standardized and needs more careful operationalization and justification.

Response: As in 1, the revision incorporates the research frontier, such as Peng (2011) and Peng (2020) to demonstrate the validity of synchronic intensity and diachronic thickness. Peng (2011) investigated the synchronic intensity and diachronic thickness of some morphemes and constructions in Chinese. The former includes “*shi* (时)” while the latter covers the competition between two non-structural strings of “yin (因)+er (而)” and “yin (因)+yi (以)”. Moreover, Peng (2020) further elaborated on two issues about critical frequency (i.e., synchronic intensity and diachronic thickness): (1) whether there exists positive correlation relationship between general frequency and critical frequency; (2) what is the minimum of critical frequency to conventionalize pragmatic implicature? These studies have examined the validity of synchronic intensity and diachronic thickness. All the above can be seen in Line 136-146 in the revision.

6. Comment: Conceptual and Theoretical Clarity. The discussion on subjectification and grammatical metaphor, although relevant, appears as an add-on rather than being fully integrated with the data analysis.

Response: The study of the critical frequency can be considered as the integrating study of frequency and mechanism of grammaticalization. Traugott (2010) held that subjectification is the subtype of the mechanism of syntactic change. In the paper, the critical frequency (i.e., synchronic intensity and diachronic thickness) of *be going to* has been determined. Therefore, the relationship between the grammaticalization of *be going to* and the mechanism of grammaticalization of *be going to* needs further exploration. In the revision, subjectification in grammaticalization is rigorously demonstrated. Firstly, the dimensions along which subjectification in grammaticalization operates raised in Traugott (1995) is introduced. Then, how the diachronic change of *be going to* meets those dimensions is demonstrated respectively. Therefore, the diachronic change of *be going to* belongs to subjectification in grammaticalization. Then the relationship between subjectification, grammaticalization and grammatical metaphor is accordingly discussed. All the above can be seen in Line 422-451 in the revision.

Editor

1. Comment: Clarity and Structure. The introduction and discussion lack focus. Clearly define the research gap and how your study addresses it. Restructure these sections to improve coherence and emphasize the study’s significance.

Response: In Introduction of the revision, the necessity of applying critical frequency in grammaticalization study is reinforced. It is carried out by the following steps: (1) limitations of the traditional frequency theory; (2) proposal of the critical frequency; (3) characteristics of the critical frequency; (4) measurement of the critical frequency; (5) significance of the critical frequency to grammaticalization study, especially the corpus-based study of grammaticalization.

In Discussion of the revision, the significance of subjectification to grammaticalization of *be going to* is highlighted. The study of the critical frequency can be taken as the integrating study of frequency and mechanism of grammaticalization. As Traugott (2010) put that subjectification is the subtype of the mechanism of syntactic change, the relationship between the grammaticalization of *be going to* and the subjectification of *be going to* needs further exploration.

2. Comment: Methodology. The experimental design and statistical methods need more detail and justification.

Response: In revision, the following parts are strengthened: (1) in Line 360-361, the application of Chi-square test is made clear; (2) in Line 395-399, the application of linear regression test is made clear; (3) in Line 404-408, the results from linear regression test are expounded.

3. Comment: Data Presentation. Figures and tables are unclear.

Response: In revision, the following parts are strengthened: (1) about Fig 1 and Table 1. It is made clear in the revision that the data in Fig 1 are from Table 1. And the data in Table 1 are explained in Line 273-275; (2) Example (17) and (18) are explained in Line 349-353; (3) Supporting Information Files describe the sources of the data and how they are obtained in Tables and Figs of the manuscript.

4. Comment: Literature Review. The manuscript requires a more thorough review of recent studies to better contextualize your findings within the field.

Response: In Line 67-77 of the revision, the studies of the grammaticalization of *be going to* are reviewed. The existing studies are categorized into 6 aspects. After identifying the limitations of existing studies, the necessity of this study is highlighted.

Note: Places that have been revised are highlighted in blue in the revision.
