# Supplementary material for: Revisiting the grammaticalization of future be going to: A corpus-based approach
Source: PLoS One. 2026 Jul 24;21(7):e0352674. doi: 10.1371/journal.pone.0352674 (PMC13399480; doi:10.1371/journal.pone.0352674)
Supplement: Attachment — Submitted filename: Response_to_Reviewers_auresp_3.docx [file pone.0352674.s009.docx]

**Manuscript Review Report**

**Title:** *Revisiting the Grammaticalization of Future be going to: A Corpus-based Approach*

**Overall Evaluation**

The manuscript presents a well-focused and relevant corpus-based investigation into the grammaticalization of the future construction *be going to*. The topic is theoretically grounded and contributes meaningfully to ongoing discussions in grammaticalization studies and corpus linguistics. The data selection, analytical orientation, and discussion are generally sound. With minor revisions—particularly to the abstract and a small methodological stylistic adjustment—the manuscript will be suitable for publication.

**Strengths**

- The study addresses an established yet still productive area of grammaticalization research.
- The corpus-based approach is appropriate and strengthens the empirical validity of the findings.
- The literature review is adequate and engages with key theoretical positions.
- The analysis and discussion are coherent and well-argued.
- Conclusions are consistent with the data and theoretical framework adopted.

**Major Comment**

**1. Abstract (Revision Required)**

The abstract needs to be revisited to meet standard academic requirements. At present, it does not fully reflect the scope and contribution of the study. It is recommended that the abstract explicitly include the following elements:

- **Research background/context** (brief positioning within grammaticalization studies)
- **Clear research objective(s)**
- **Data source(s)** (type of corpus, size, period, or variety, where relevant)
- **Methodological approach** (corpus-based/quantitative–qualitative analysis)
- **Key findings/results**
- **Main contribution/implications** of the study

The revised abstract should be concise, informative, and reflective of what the study actually achieves.

**Minor Comment**

**2. Methodology Section (Stylistic Adjustment)**

In the methodology section, the use of **future tense** (e.g., “will be analyzed,” “will be examined”) should be avoided. Instead, the section should consistently employ:

- **Past tense** (to describe procedures already carried out), or
- **Present simple tense** (to describe standard methodological practices).

This change will improve academic style and align the manuscript with conventional research-writing norms.

**Language and Style**

The language is generally clear and academic. No major grammatical or stylistic issues were noted beyond the tense usage mentioned above.

**Recommendation**

**Accept with minor revisions**

Once the abstract is revised to include essential components, keywords are added, and the methodology tense is corrected, the manuscript will be ready for publication.
