# Supplementary material for: Revisiting the grammaticalization of future be going to: A corpus-based approach
Source: PLoS One. 2026 Jul 24;21(7):e0352674. doi: 10.1371/journal.pone.0352674 (PMC13399480; doi:10.1371/journal.pone.0352674)
Supplement: Attachment — Submitted filename: Response_to_Reviewers_auresp_4.docx [file pone.0352674.s012.docx]

Rebuttal Letter

We sincerely appreciate the editor and reviewers for their thoughtful feedback and constructive suggestions, which have greatly helped improve our manuscript. The following are one-to-one responses to the comments and suggestions.

Reviewers Comments

Reviewer 3

1. Comment: The abstract needs to be revisited to meet standard academic requirements.

Response: The revision gives full consideration of the suggestion, including research background, research objectives, data sources, methodological approach, key findings and main contribution of the study.

2. Comment: Stylistic Adjustment. In the methodology section, the section should consistently employ past tense or present simple tense.

Response: The revision employs the present simple tense in the methodology section to align the manuscript with conventional norms.

Reviewer 4

1. Comment: Argumentation and Theoretical Balance. “The tone could be moderated slightly to acknowledge more explicitly what general frequency studies have successfully explained.”

Response: The contribution of general frequency to grammaticalization is manifested in Line 59-61. The revision not only clarifies the significance of frequency to grammaticalization, but also highlights the quantitative research orientation, thus informing the methodological approach adopted in this study.

2. Comment: “some examples (e.g., highly marked or violent sentences) may raise stylistic concerns for certain journals; consider whether neutral alternatives could be substituted without loss of analytical value.”

Response: In Line 210 and Line 214, example (7) and (10) are substituted to avoid any possible stylistic concerns.

Note: Places that have been revised are highlighted in blue in the revision.
