# Supplementary material for: Revisiting the grammaticalization of future be going to: A corpus-based approach
Source: PLoS One. 2026 Jul 24;21(7):e0352674. doi: 10.1371/journal.pone.0352674 (PMC13399480; doi:10.1371/journal.pone.0352674)
Supplement: Attachment — Submitted filename: Response_to_Reviewers_auresp_5.docx [file pone.0352674.s013.docx]

Rebuttal Letter

We sincerely appreciate the editor and reviewers for their thoughtful feedback and constructive suggestions, which have greatly helped improve our manuscript. The following are one-to-one responses to the comments and suggestions.

Reviewers Comments

1. Comment: The paper is overly long and occasionally repetitive.

Response:

We thank the reviewer for this constructive comment. We agree that the original manuscript was unnecessarily lengthy and contained some redundancy. In response, we have substantially shortened and streamlined the manuscript.

Specifically, we have made the following changes:

(1) The detailed corpora descriptions have been removed from the Abstract, as this information is already fully presented in the Methodology section.

(2) Throughout the paper, identical or similar expressions have been integrated and streamlined to avoid repetition and enhance conciseness, as will be shown in the next Comment and Response.

We believe the revised manuscript is now more concise, readable, and free of unnecessary repetition, while retaining all essential information and methodological transparency. We hope that the revised version now meets the journal’s standards for length and clarity.

2. Comment: The literature review could be streamlined.

Response:

We thank the reviewer for this helpful suggestion. We agree that the literature review in the original submission was longer than necessary and included some repetitive references. In response, we have thoroughly revised this section to make it more focused, concise, and directly relevant to the study’s objectives.

Specifically, we have removed references that provided only general background or were only loosely related to our specific research question. For example:

(1) In the previous draft, the premise of grammaticalization by Peng [21] appeared in both the “Context” section and the “Critical Context and Critical Frequency” section. In the current draft, this reference has been retained only in the latter to eliminate redundancy and focus on the specific topic.

(2) Similarly, Critical Frequency Hypothesis by Peng [21] (i.e., which posits that providing the critical frequency accumulates a considerable diachronic thickness and reaches a certain synchronic intensity, grammaticalization may be triggered.) was previously cited in both the Introduction and the literature review. In the present draft, it has been removed from the literature review while remaining in the Introduction.

We believe these revisions have resulted in a more streamlined literature review that is easier to follow and better aligned with the journal’s expectations for concise yet comprehensive background information. All essential prior work remains appropriately cited, and the logical link to our study’s rationale is now clearer.

3. Comment: The distinction between critical frequency and general frequency is asserted but not rigorously justified.

Response:

We thank the reviewer for this insightful comment. In the original manuscript, the distinction between critical frequency and general frequency may not have been sufficiently justified. To address this concern, we have now provided a more rigorous rationale from the following aspects:

(1) Theoretically, not all frequencies observed during the grammaticalization process of a given grammaticalizing item can trigger grammaticalization. Heine [19] and Diewald [20] concurrently introduced the concept of “critical context”, in which the frequencies observed are accordingly termed “critical frequencies”. Peng [21] further distinguished two types of frequencies of the grammaticalizing items, i.e., critical frequency and non-critical frequency. Critical frequency is independent of general frequency, in that an increase of the former may (but not necessarily) accompany an increase of the latter.

(2) Empirically, Peng [21] quantitatively investigated the critical frequency of selected morphemes and constructions in Chinese by the application of corpora. Peng [35] further explored two issues concerning critical frequency: (1) whether there exists a positive correlation between general frequency and critical frequency; (2) what the minimum critical frequency required for pragmatic implicature to become conventionalized is.

(3) In the present paper, we focus our research on two issues: ① the critical frequency of future *be going to*; ② the relationship between its critical frequency and general frequency. Since both issues have been exhaustively examined in the manuscript, we will not reiterate the detailed findings here.

We believe this expounded rationale clarifies the theoretical and empirical basis for distinguishing critical frequency from general frequency and properly situates our research within the existing literature.

4. Comment: The criteria for identifying “critical contexts” are not replicable.

Response:

We thank the reviewer for raising this important point regarding replicability. We agree that, in principle, replicability may not be an inherent or necessary attribute of critical contexts across all cases, given that different morphemes or constructions may involve distinct critical contexts, and any case that triggers grammaticalization may be considered valid. In fact, as mentioned in our paper, “A linguistic form may be associated with a number of different critical contexts”. Nevertheless, for the specific case of *be going to*, we have taken concrete steps to ensure replicability. Specifically, we formalized the critical context through explicit logical reasoning and conducted an exhaustive corpus retrieval, which enabled us to determine its critical frequency in an objective and transparent manner. Consequently, the identification of the critical context for the grammaticalization of *be going to* in our study is fully replicable.

5. Comment: The classification of verbs into three types appears ad hoc.

Response:

We thank the reviewer for this comment. We would like to clarify that the threefold classification of verbs in our study is not *ad hoc* but is systematically derived from the three distinct contexts involved in the grammaticalization of *be going to*, namely the initial stage, the critical context, and the isolating context (see Lines 117–134).

In the initial stage, grammaticalization is not yet triggered. Here, *be going to* exclusively denotes movement in space and therefore co-occurs only with action verbs. In the critical context, ambiguity arises, allowing *be going to* to refer either to spatial movement or future time. In the isolating context, as noted in Line 128, “the isolating context is incompatible with some salient properties of the source meaning”; consequently, *be going to* refers unambiguously to future time and can be followed by mental or stative verbs.

Correspondingly, verbs following future *be going to* naturally fall into three categories:

(1) Verbs in the initial stage. These are action verbs that occur in both *go and V* and *go to V* constructions and do not cause ambiguity. They are empirically retrievable from corpora.

(2) Verbs in the isolating context. These are mental or stative verbs. They are the indicator of the full grammaticalization of *be going to*. The identification of this type of words follows the well-established criteria of Halliday & Matthiessen [39].

(3) Verbs in the critical context. These verbs have the potential to trigger grammaticalization of *be going to*. They are derived by subtracting the first two categories from the total set of verbs attested during the grammaticalization process of *be going to*.

The complete procedure for obtaining these three categories is detailed in Lines 391–401 of the revised manuscript.

Methodologically, all the verbs appearing during the whole grammaticalization process of future *be going to* have been exhaustively retrieved, with no exceptions. Theoretically, the identification of the 159 words is strictly justified with reference to well-established or independently verified criteria, including the grammaticalization process of *go and V* to *go to V*, as well as the framework proposed by Halliday & Matthiessen [39].

Therefore, far from being *ad hoc*, our classification is grounded in both rigorous methodology and sound theoretical justification.

6. Comment: No clear annotation protocol or inter-rater reliability is provided.

Response:

Thank you for this comment. We would like to clarify that the present study does **not** involve any manual annotation or subjective classification by human coders. Therefore, an annotation protocol or inter-rater reliability (IRR) is not applicable to our methodology.

Specifically, all data in this study are obtained through the following approaches:

**(1) Exhaustive corpus retrieval** using formalized search queries based on explicit linguistic criteria. The study is built on large, balanced, and authoritative diachronic corpora, with corpus sizes reported in the manuscript. Exhaustive retrieval—rather than random sampling—was performed using predefined search queries, ensuring the replicability of both the retrieved data and the results.

**(2) Logical determination of critical contexts**, as illustrated by the grammaticalization of *be going to*. With ambiguity as its distinctive features, the critical context is rigorously defined (including the procedure for identifying the 159 words). The critical frequency is derived through logical reasoning, independent of any subjective judgment or inter-rater input. The formalization of the critical context and the formulation of search queries remain consistent regardless of the researcher involved.

All retrieved data are presented as standardized frequencies per million words to ensure comparability across texts of different sizes or from different historical periods.

Since no manual annotation or subjective classification was performed—i.e., the preconditions for requiring an annotation protocol and IRR are absent— we respectfully maintain that such procedures are not necessary for this study. We hope this clarification addresses the concern. Thank you again for your careful reading.

7. Comment: The leap from correlation to “threshold triggering grammaticalization” is theoretically overstated.

Response:

Thank you for this constructive comment. We acknowledge that our use of the so-called “threshold triggering” may inadvertently imply a stronger causal claim than intended. In response, we have revised our interpretation as follows:

(1) The concept of “critical frequency,” operationalized in this study as diachronic thickness and synchronic intensity, is a central focus of our investigation into the grammaticalization of *be going to*. We suggest that it is critical frequency, rather than general frequency, that plays a more direct role in facilitating grammaticalization. In this sense, critical frequency can be seen as a driving force behind the process. The term “threshold” is used heuristically and metaphorically to suggest that grammaticalization may require a certain level of critical frequency to take effect.

(2) The procedures for determining diachronic thickness and synchronic intensity are thoroughly and rigorously detailed in the present study.

(3) Additionally, a linear regression test was conducted to examine the relationship between general frequency and critical frequency. The results show a strong correlation between the two for *be going to*.

(4) Given this correlation, we do **not** claim a direct causal leap from correlation to the proposed “threshold triggering.” Instead, we treat the threshold metaphor as a descriptive heuristic for understanding how critical frequency may contribute to grammaticalization, rather than positing a discrete triggering event.

We hope that these have adequately addressed the concern by clarifying the theoretical status of “threshold” and avoiding any overstatement of causality.

8. Comment: No validation or manual checking procedures are reported.

Response:

We thank the reviewer for raising this important point. We agree that validation and manual checking are essential, even for exhaustive corpus queries. In the original manuscript, we did not report such procedures, as this study did not employ statistical models (e.g., classifiers) that require manually annotated training data. However, in response to the reviewer’s comment, we have conducted a post-hoc manual validation.

(1) Verification criteria: All 159 retrieved instances were manually examined against the predefined linguistic criteria, such as that by Halliday & Matthiessen [39]. Specifically, we verified whether each instance truly conforms to the formalized definition of the target category (e.g., the critical context of *be going to*).

(2) Accuracy assessment: The number of correctly retrieved instances was counted, and the accuracy rate was calculated as [correct instances / total instances × 100%]. The observed accuracy was 99.37%, indicating that our search queries and retrieval procedures are highly reliable.

This manual checking confirms that the vast majority of the retrieved data meet the established criteria, and the results reported in this study are robust. We hope this addresses the reviewer’s concern.

9. Comment: Improve statistical rigor and interpretation.

Response:

We thank the reviewer for this valuable suggestion. We have improved the statistical rigor and interpretation of our results accordingly. Specifically, we have clarified the linear regression analysis examining the relationship between critical frequency (as the independent variable, *x*) and general frequency (as the dependent variable, *y*).

(1) The regression model reveals a strong positive linear relationship between $x$ and $y$, with the fitted equation $y=10.046x+0.08$.

(2) The coefficient of determination $R^{2}=0.984$ indicates that 98.4% of the variance in $y$ is accounted for by $x$. This high value reflects an excellent model fit and suggests that the two frequency measures are very strongly associated.

(3) The slope ($10.046$) indicates a directly proportional relationship: a one-unit increase in *x* corresponds to an increase of approximately 10.046 units in *y*. The intercept ($0.08$) estimates the value of $y$ when $x$ is zero.

We emphasize that this regression analysis is intended to quantify the strength of the linear association between the two frequency measures, rather than to claim a causal direction. These results have been clearly described in the revised manuscript (see Lines 412—418). We believe these revisions address the reviewer’s concern regarding statistical rigor and interpretation. Thank you for your careful reading.

10. Comment: Substantially revise language and structure.

Response:

We have substantially revised the language and structure throughout the manuscript in response to this comment. The revision was carried out by the author in collaboration with professional academic editors to ensure idiomaticity and overall readability. To provide a concrete example, the paragraph originally located in Lines 250—253 has been restructured in Lines 174—177 to achieve greater logical fluency.

We hope that the revised manuscript now meets the language standards expected by PLOS ONE.

Note: Places that have been revised are highlighted in blue in the revision.
