# Supplementary material for: Revisiting the grammaticalization of future be going to: A corpus-based approach
Source: PLoS One. 2026 Jul 24;21(7):e0352674. doi: 10.1371/journal.pone.0352674 (PMC13399480; doi:10.1371/journal.pone.0352674)
Supplement: Peer review history

# Peer review: Decision Letter 0

**DOI:** 10.1371/journal.pone.0352674.r001

**Authors:** Dawit Dibekulu

2 Jul 2025

PONE-D-25-26551

Revisiting the Grammaticalization of Future be going to: A Corpus-based Approach

PLOS ONE

Dear Dr. Dai,

Thank you for submitting your manuscript to PLOS ONE. After careful consideration, we feel that it has merit but does not fully meet PLOS ONE’s publication criteria as it currently stands. Therefore, we invite you to submit a revised version of the manuscript that addresses the points raised during the review process.

Please submit your revised manuscript by Aug 16 2025 11:59PM. If you will need more time than this to complete your revisions, please reply to this message or contact the journal office at plosone@plos.org. When you're ready to submit your revision, log on to https://www.editorialmanager.com/pone/ and select the 'Submissions Needing Revision' folder to locate your manuscript file.

Please include the following items when submitting your revised manuscript:

- A rebuttal letter that responds to each point raised by the academic editor and reviewer(s). You should upload this letter as a separate file labeled 'Response to Reviewers'.
- A marked-up copy of your manuscript that highlights changes made to the original version. You should upload this as a separate file labeled 'Revised Manuscript with Track Changes'.
- An unmarked version of your revised paper without tracked changes. You should upload this as a separate file labeled 'Manuscript'.

If you would like to make changes to your financial disclosure, please include your updated statement in your cover letter. Guidelines for resubmitting your figure files are available below the reviewer comments at the end of this letter.

If applicable, we recommend that you deposit your laboratory protocols in protocols.io to enhance the reproducibility of your results. Protocols.io assigns your protocol its own identifier (DOI) so that it can be cited independently in the future. For instructions see: https://journals.plos.org/plosone/s/submission-guidelines#loc-laboratory-protocols. Additionally, PLOS ONE offers an option for publishing peer-reviewed Lab Protocol articles, which describe protocols hosted on protocols.io. Read more information on sharing protocols at https://plos.org/protocols?utm_medium=editorial-email&utm_source=authorletters&utm_campaign=protocols.

We look forward to receiving your revised manuscript.

Kind regards,

Dawit Dibekulu, PhD

Academic Editor

PLOS ONE

Journal Requirements:

When submitting your revision, we need you to address these additional requirements.

1. Please ensure that your manuscript meets PLOS ONE's style requirements, including those for file naming. The PLOS ONE style templates can be found at

https://journals.plos.org/plosone/s/file?id=wjVg/PLOSOne_formatting_sample_main_body.pdf and

https://journals.plos.org/plosone/s/file?id=ba62/PLOSOne_formatting_sample_title_authors_affiliations.pdf

2. We note that the grant information you provided in the ‘Funding Information’ and ‘Financial Disclosure’ sections do not match.

When you resubmit, please ensure that you provide the correct grant numbers for the awards you received for your study in the ‘Funding Information’ section.

3. Thank you for stating the following financial disclosure: [This research is supported by The Social Science Funding Project of Hunan Provincegrant number 21YBA074and The Key Project from Education Department of Hunan Provincegrant number 21A0209].

Please state what role the funders took in the study.  If the funders had no role, please state: ""The funders had no role in study design, data collection and analysis, decision to publish, or preparation of the manuscript.""

If this statement is not correct you must amend it as needed.

Please include this amended Role of Funder statement in your cover letter; we will change the online submission form on your behalf.

4. We note that your Data Availability Statement is currently as follows: [All relevant data are within the manuscript and its Supporting Information files.]

Please confirm at this time whether or not your submission contains all raw data required to replicate the results of your study. Authors must share the “minimal data set” for their submission. PLOS defines the minimal data set to consist of the data required to replicate all study findings reported in the article, as well as related metadata and methods (https://journals.plos.org/plosone/s/data-availability#loc-minimal-data-set-definition).

For example, authors should submit the following data:

- The values behind the means, standard deviations and other measures reported;

- The values used to build graphs;

- The points extracted from images for analysis.

Authors do not need to submit their entire data set if only a portion of the data was used in the reported study.

If your submission does not contain these data, please either upload them as Supporting Information files or deposit them to a stable, public repository and provide us with the relevant URLs, DOIs, or accession numbers. For a list of recommended repositories, please see https://journals.plos.org/plosone/s/recommended-repositories.

If there are ethical or legal restrictions on sharing a de-identified data set, please explain them in detail (e.g., data contain potentially sensitive information, data are owned by a third-party organization, etc.) and who has imposed them (e.g., an ethics committee). Please also provide contact information for a data access committee, ethics committee, or other institutional body to which data requests may be sent. If data are owned by a third party, please indicate how others may request data access.

Additional Editor Comments:

Please address all the comments and follow author guide line.

[Note: HTML markup is below. Please do not edit.]

Reviewers' comments:

Reviewer's Responses to Questions

**Comments to the Author**

1. Is the manuscript technically sound, and do the data support the conclusions?

The manuscript must describe a technically sound piece of scientific research with data that supports the conclusions. Experiments must have been conducted rigorously, with appropriate controls, replication, and sample sizes. The conclusions must be drawn appropriately based on the data presented.

Reviewer #1: Yes

Reviewer #2: Partly

**********

2. Has the statistical analysis been performed appropriately and rigorously?

Reviewer #1: Yes

Reviewer #2: No

**********

3. Have the authors made all data underlying the findings in their manuscript fully available?

The PLOS Data policy requires authors to make all data underlying the findings described in their manuscript fully available without restriction, with rare exception (please refer to the Data Availability Statement in the manuscript PDF file). The data should be provided as part of the manuscript or its supporting information, or deposited to a public repository. For example, in addition to summary statistics, the data points behind means, medians and variance measures should be available. If there are restrictions on publicly sharing data—e.g. participant privacy or use of data from a third party—those must be specified.

Reviewer #1: Yes

Reviewer #2: Yes

**********

4. Is the manuscript presented in an intelligible fashion and written in standard English?

PLOS ONE does not copyedit accepted manuscripts, so the language in submitted articles must be clear, correct, and unambiguous. Any typographical or grammatical errors should be corrected at revision, so please note any specific errors here.

Reviewer #1: Yes

Reviewer #2: No

**********

5. Review Comments to the Author

Please use the space provided to explain your answers to the questions above. You may also include additional comments for the author, including concerns about dual publication, research ethics, or publication ethics. (Please upload your review as an attachment if it exceeds 20,000 characters)

Reviewer #1: This manuscript presents an interesting and relevant exploration into the critical frequency, synchronic intensity, and diachronic thickness of the future construction "be going to". The focus on corpus-based analysis using EEBO, COCA, and Google Books provides a robust methodological foundation for investigating linguistic change. The clear articulation of the research's purpose—to clarify the relationship between critical and general frequencies—is a significant strength. The paper is well-structured, with a logical flow from introduction to methodology, findings, and discussion.

Strengths:

Originality and Significance: The research addresses a pertinent area in grammaticalization studies by focusing on the concept of "critical frequency," which offers a new perspective beyond traditional general frequency analysis. The findings regarding the diachronic thickness (110 years, 1580-1690) and synchronic intensity (0.81 per million words) of "be going to" are specific and contribute valuable empirical data to the field. The proposed six-stage grammaticalization process of "be going to" is clearly delineated and supported with examples .

Methodological Rigor: The use of large-scale historical corpora such as EEBO (755 million words, 1470s-1690s) and Google Books (200 billion words, 1500-2000) strengthens the empirical claims. The detailed explanation of search queries (SQ1, SQ2, SQ3, SQ4) and verb categorization demonstrates a systematic approach to data retrieval . The inclusion of tables (Table 1 and Table 2) effectively presents the frequency data, illustrating the diachronic changes and the competition among candidate future expressions (e.g., "be going to" vs. "be coming to" vs. "be leaving to").

Clarity and Intelligibility: The manuscript is generally well-written in standard English, and the complex concepts of grammaticalization, critical context, and critical frequency are explained intelligibly .

Points to be reconsidered:

Definition of Critical Context and Ambiguity: While the paper defines critical context as having the potential to evoke ambiguity , it states that "it is hard to evaluate the ambiguity by quantitative methods" and that "the degree of ambiguity they cause forms a cline". For a study heavily reliant on quantitative corpus analysis, a more detailed discussion or qualitative illustration of how ambiguity was identified and verbs categorized into critical contexts would enhance the rigor. Providing specific examples of sentences where "be going to" initially caused ambiguity in the critical context (beyond the general examples in 1b and 1c on page 1 ) would be beneficial.

Discussion on "Go" Winning the Competition: The paper states that "the frequency of going overwhelms others, which makes it the only chosen form" in the competition for future expression. While frequency is presented as the crucial factor, a deeper discussion on why "go" had a higher frequency compared to "come" and "leave" in contexts leading to grammaticalization would strengthen the argument. Were there semantic, pragmatic, or structural advantages of "go" that contributed to its higher frequency in these specific contexts? (Pages 7-8, particularly Table 2, show the frequency data, but the explanation could be expanded).

Integration of Criteria: The manuscript mentions the exclusion of mental verbs like "recall, resolve, hate" as indicators of full grammaticalization. It would be helpful to explicitly illustrate how these verbs, or similar examples, demonstrate the "isolating context" (where the target meaning is favored and distinct from the source meaning) , linking this back to the categorization of verbs for data retrieval.

Future Exploration: The statement that "The finer evaluation of the ambiguity they cause will call for further exploration" is noted. While understandable, given the scope, outlining specific avenues for such future exploration could demonstrate a clearer path forward for this line of research.

Reviewer #2: The paper addresses an important and enduring question in grammaticalization theory by attempting to operationalize and empirically validate the notion of "critical frequency" through diachronic corpus analysis. The effort to correlate general and critical frequency using large corpora (EEBO, COCA, Google Books) is commendable and aligns well with current trends in data-driven historical linguistics. However, several critical issues regarding statistical rigor, clarity of language, and methodological transparency need to be addressed to bring this manuscript to publishable standard.

1. Technical Soundness and Data-Conclusion Alignment

While the theoretical framing is grounded in a substantial body of literature and the corpus-based methodology is generally suitable, the data do not fully support the strong conclusions drawn. The key construct—critical frequency—is not defined with adequate statistical or methodological precision. The transition between descriptive statistics and theoretical claims is often abrupt, and there is little attempt to validate findings beyond raw frequency counts.

2. Statistical Analysis

Although the manuscript uses chi-square testing and linear regression to support its claims (e.g., correlation between general and critical frequency), several shortcomings weaken the statistical rigor:

No confidence intervals, standard errors, or effect sizes are reported.

Assumptions behind the regression model (e.g., normality, homoscedasticity) are not discussed.

The classification of verbs into "critical" and "non-critical" contexts is not empirically tested, nor is any interrater reliability or objective metric used.

The lack of inferential statistical thresholds for defining "critical frequency" raises concerns about replicability.

Hence, while some statistical tools are employed, the analytical framework is incomplete and requires substantial reinforcement.

3. Data Availability

The manuscript is compliant with PLOS ONE’s data policy. All corpora used are publicly accessible, and the frequency data are transparently presented in the tables and figures. The data availability statement is adequate.

4. Language and Presentation

The manuscript is not written in standard academic English. There are frequent issues with grammar, syntax, and idiomatic phrasing. Phrasings such as "what is to be mentioned here is that…" and “may be used in whatever context” are awkward or ambiguous. These detract from the intelligibility of otherwise important points. A thorough language revision by a professional academic editor or native English speaker is strongly recommended before resubmission.

5. Conceptual and Theoretical Clarity

While the authors make an ambitious attempt to connect frequency, grammaticalization, and subjectification, several theoretical points lack coherence:

The six-stage model of grammaticalization is useful but under-supported by empirical evidence beyond surface frequency patterns.

The definition of “synchronic intensity” and “diachronic thickness” is not universally standardized and needs more careful operationalization and justification.

The discussion on subjectification and grammatical metaphor, although relevant, appears as an add-on rather than being fully integrated with the data analysis.

6. Recommendations for Improvement

To strengthen this manuscript, I suggest the following:

Introduce a more rigorous statistical framework to define and test “critical frequency.”

Report confidence intervals and variance measures throughout.

Rework the language for clarity, precision, and academic tone.

Ensure that key theoretical terms are clearly defined and consistently applied.

Provide more nuanced interpretations of corpus findings, including discussion of false positives and semantic ambiguity.

Conclusion:

The manuscript has potential due to its innovative focus and use of extensive diachronic corpora, but it requires substantial revisions in methodology, statistical rigor, and language to meet the standards expected in a journal like PLOS ONE. I encourage the authors to carefully address these issues, as doing so will significantly improve the quality, credibility, and impact of the work.

**********

6. PLOS authors have the option to publish the peer review history of their article (what does this mean?). If published, this will include your full peer review and any attached files.

If you choose “no”, your identity will remain anonymous but your review may still be made public.

**Do you want your identity to be public for this peer review?**  For information about this choice, including consent withdrawal, please see our Privacy Policy.

Reviewer #1: **Yes:** EBA TERESA GAROMA

Reviewer #2: **Yes:** Mohanad Aljbour

**********

[NOTE: If reviewer comments were submitted as an attachment file, they will be attached to this email and accessible via the submission site. Please log into your account, locate the manuscript record, and check for the action link "View Attachments". If this link does not appear, there are no attachment files.]

While revising your submission, please upload your figure files to the Preflight Analysis and Conversion Engine (PACE) digital diagnostic tool, https://pacev2.apexcovantage.com/. PACE helps ensure that figures meet PLOS requirements. To use PACE, you must first register as a user. Registration is free. Then, login and navigate to the UPLOAD tab, where you will find detailed instructions on how to use the tool. If you encounter any issues or have any questions when using PACE, please email PLOS at figures@plos.org. Please note that Supporting Information files do not need this step.

# Author comment: Author response to Decision Letter 1

**DOI:** 10.1371/journal.pone.0352674.r002

20 Aug 2025

Rebuttal Letter

We sincerely appreciate the editor and reviewers for their thoughtful feedback and constructive suggestions, which have greatly helped improve our manuscript. The following are one-to-one responses to the comments and suggestions.

Reviewer Comments

Reviewer 1

1. Comment: Definition of Critical Context and Ambiguity. “Providing specific examples of sentences where ‘be going to’ initially cause ambiguity in the critical context would be beneficial”.

Response: In the six stages of the grammaticalization of future be going to, the author gives more examples to explain the grammaticalization in detail, especially how ambiguity arises in the critical context. The revised paper reads as “In Example (7), be going to is followed by an action verb kill, and be going to here has two interpretations. The first one is ‘I am moving from one place to another with the aim of killing the president’ while the second one is ‘I will kill the president in the future’. Thus, ambiguous interpretations arise, which forms the critical context for the grammaticalization of be going to.” In the critical context, the pragmatic inference can be fulfilled. be going to, originally meaning movement in space, can refer to future in time by means of metaphor. The critical context provides room to trigger grammaticalization.

2. Comment: Discussion on “go” Winning the Competition. “a deeper discussion on why ‘go’ had a higher frequency compared to ‘come’ and ‘leave’ in contexts leading to grammaticalization would strengthen the argument”.

Response: The three verbs of go, come and leave are unmarked for the manner of movement, that is, they do not specify the detail of movement. All of them have the potential to grammaticalize into future markers. In the revision, the reason why come and leave are not grammaticalized are explained. As for leave, “the frequency of leave is too low to be further grammaticalized.” As for come, “out of the top 10 high-frequency words, 5 coincide with those from SQ2. They are take (with its frequency of 23), see (20), meet (15), be (14) and make (12). The fact means that there does exist critical context for the potential grammaticalization of come into a future marker. But the relative low frequency has not yet reached the threshold for the grammaticalization of come.” In conclusion, the high critical frequency of be going to leads to the repeated pragmatic inference, which triggers grammaticalization. The frequency of be leaving to is too low to trigger grammaticalization. The critical frequency of be coming to is not high enough to trigger grammaticalization.

3. Comment: Integration of Criteria. “It would be helpful to explicitly illustrate how these verbs, or similar examples, demonstrate the ‘isolating context’, linking this back to the categorization of verbs for data retrieval.”

Response: In this paper, we categorize three types of contexts for the investigation of the grammaticalization process of future be going to: initial stage, critical context and isolating context. The demonstration of the three types of contexts can be seen in Line 162-166. In the initial stage, grammaticalization is not triggered. be going to only means movement in space, so it is followed by action verbs. Only in the critical context, ambiguity arises. That is to say be going to can refer to either movement in space or future in time. As mentioned in Line 91, “the isolating context is incompatible with some salient properties of the source meaning”. In the isolating context, be going to only refers to future in time in all contexts. Be going to can be followed by verbs of mental experience or stative verbs.

Correspondingly, verbs can be classified into three categories. (1) verbs in initial stage: these verbs will not cause ambiguity; (2) verbs in critical context: these verbs will cause ambiguity; (3) verbs in isolating context: mental verbs or stative verbs. How to get these three categories of verbs is demonstrated in Line 358-368 of the revised paper.

4. Comment: Future Exploration. “outlining specific avenues for such future exploration could demonstrate a clearer path forward for this line of research.”

Response: In the revision, Section “for Further Research” is added to give the two topics in future research. The generality of critical frequency will be the focus of future exploration. It includes the following two topics. The first one is the generality of critical frequency across languages; the second one is the transcategory (across morphemes and constructions) generality of critical frequency.

Reviewer 2

1. Comment: Technical Soundness and Data-Conclusion Alignment. “The key construct—critical frequency—is not defined with adequate statistical or methodological precision.”

Response: The revision incorporates the research frontier, such as Peng (2011) and Peng (2020) to demonstrate the validity of critical frequency. Peng (2011) investigated the critical frequency of some morphemes and constructions in Chinese. By determining the critical frequency as well as the general frequency of these morphemes and constructions, Peng (2011) proved the validity of critical frequency in grammaticalization. Peng (2020) further explored two issues about critical frequency: (1) whether there exists positive correlation relationship between general frequency and critical frequency; (2) what is the minimum of critical frequency to conventionalize pragmatic implicature? All these works prove the validity of critical frequency theory.

2. Comment: Conceptual and Theoretical Clarity. “The six-stage model of grammaticalization is useful but under-supported by empirical evidence beyond surface frequency patterns.”

Response: In the revision, the six stages of grammaticalization of future be going to are expounded in detail respectively. In Line 153-169, the morphosyntactic characteristics of the six stages are demonstrated. Especially in the fifth stage, the initial stage, the critical context and the isolating context are further manifested. In Line 170-184, examples from the corpus are given to each stage. In Line 185-218, each example is explained in detail. By doing so, the six stages of grammaticalization of future be going to are demonstrated by empirical evidence.

3. Comment: Conceptual and Theoretical Clarity. The definition of “synchronic intensity” and “diachronic thickness” is not universally standardized and needs more careful operationalization and justification.

Response: As in 1, the revision incorporates the research frontier, such as Peng (2011) and Peng (2020) to demonstrate the validity of synchronic intensity and diachronic thickness. Peng (2011) investigated the synchronic intensity and diachronic thickness of some morphemes and constructions in Chinese. The former includes “shi (时)” while the latter covers the competition between two non-structural strings of “yin (因)+er (而)” and “yin (因)+yi (以)”. Moreover, Peng (2020) further elaborated on two issues about critical frequency (i.e., synchronic intensity and diachronic thickness): (1) whether there exists positive correlation relationship between general frequency and critical frequency; (2) what is the minimum of critical frequency to conventionalize pragmatic implicature? All the works prove the validity of synchronic intensity and diachronic thickness. All the above can be seen in Line 116-125 in the revision.

4. Comment: The discussion on subjectification and grammatical metaphor, although relevant, appears as an add-on rather than being fully integrated with the data analysis.

Response: In the revision, subjectification in grammaticalization is rigorously demonstrated. Firstly, the dimensions along which subjectification in grammaticalization operates raised in Traugott (1995) is introduced. Then, how the diachronic change of be going to meets those dimensions is demonstrated respectively. Therefore, the diachronic change of be going to belongs to subjectification in grammaticalization. Then the relationship between subjectification, grammaticalization and grammatical metaphor is accordingly discussed. All the above can be seen in Line 385-414 in the revision.

5. Comment: Language and Presentation.

Response: The revision has been thoroughly modified by the author and professional academic editors to guarantee its idiomaticity, which will meet the standard of PLOS ONE.

6. Comment: Statistical Analysis. Reviewer 2 raises the following questions: (1) No confidence intervals, standard errors, or effect sizes are reported; (2) Assumptions behind the regression model are not discussed; (3) any interrater reliability or objective metric is not used; (4) The lack of inferential statistical thresholds for defining “critical frequency”.

Response: As to (2), in the paper chi-square test is applied in Figure 1. Linear regression is applied to fit the data in Figure 2 and the data are homogeneously distributed. Although (1), (3) and (4) are illuminating, the present paper is to determine the relationship between the critical frequency and the general frequency and only linear regression test is used, which is only related to (2).

Note: Places that have been revised are highlighted in blue in the revision.

Attachment Submitted filename: Response to Reviewers.docx

# Editor report: Decision Letter 1

**DOI:** 10.1371/journal.pone.0352674.r003

**Authors:** Dawit Dibekulu

28 Aug 2025

PONE-D-25-26551R1

Revisiting the Grammaticalization of Future be going to: A Corpus-based Approach

PLOS ONE

Dear Dr. Dai,

Thank you for submitting your manuscript to PLOS ONE. After careful consideration, we feel that it has merit but does not fully meet PLOS ONE’s publication criteria as it currently stands. Therefore, we invite you to submit a revised version of the manuscript that addresses the points raised during the review process.

Please submit your revised manuscript by Oct 12 2025 11:59PM. If you will need more time than this to complete your revisions, please reply to this message or contact the journal office at plosone@plos.org. When you're ready to submit your revision, log on to https://www.editorialmanager.com/pone/ and select the 'Submissions Needing Revision' folder to locate your manuscript file.

Please include the following items when submitting your revised manuscript:

- A rebuttal letter that responds to each point raised by the academic editor and reviewer(s). You should upload this letter as a separate file labeled 'Response to Reviewers'.
- A marked-up copy of your manuscript that highlights changes made to the original version. You should upload this as a separate file labeled 'Revised Manuscript with Track Changes'.
- An unmarked version of your revised paper without tracked changes. You should upload this as a separate file labeled 'Manuscript'.

If you would like to make changes to your financial disclosure, please include your updated statement in your cover letter. Guidelines for resubmitting your figure files are available below the reviewer comments at the end of this letter.

If applicable, we recommend that you deposit your laboratory protocols in protocols.io to enhance the reproducibility of your results. Protocols.io assigns your protocol its own identifier (DOI) so that it can be cited independently in the future. For instructions see: https://journals.plos.org/plosone/s/submission-guidelines#loc-laboratory-protocols. Additionally, PLOS ONE offers an option for publishing peer-reviewed Lab Protocol articles, which describe protocols hosted on protocols.io. Read more information on sharing protocols at https://plos.org/protocols?utm_medium=editorial-email&utm_source=authorletters&utm_campaign=protocols.

We look forward to receiving your revised manuscript.

Kind regards,

Dawit Dibekulu, PhD

Academic Editor

PLOS ONE

Journal Requirements:

If the reviewer comments include a recommendation to cite specific previously published works, please review and evaluate these publications to determine whether they are relevant and should be cited. There is no requirement to cite these works unless the editor has indicated otherwise.

Additional Editor Comments:

Below are concise comments to the author of the manuscript submitted to PLOS ONE, summarizing key points for major revisions based on reviewer feedback and editorial assessment. These are written in a professional, direct tone suitable for an academic editor.

Comments to Authors:

Dear Authors,

Thank you for submitting your manuscript to PLOS ONE. After careful consideration of the peer reviews and my own evaluation, I have determined that your manuscript requires revisions to meet the journal’s standards. Below are the key areas for improvement:

Clarity and Structure: The introduction and discussion lack focus. Clearly define the research gap and how your study addresses it. Restructure these sections to improve coherence and emphasize the study’s significance.

Methodology: The experimental design and statistical methods need more detail and justification. Address Reviewer 2’s concerns about specific methodological issue, e.g., sample size or controls and discuss limitations transparently.

Data Presentation: Figures and tables are unclear. Improve their organization, labeling, and captions to ensure accessibility and alignment with the results.

Literature Review: The manuscript requires a more thorough review of recent studies to better contextualize your findings within the field.

Please revise the manuscript, addressing all reviewer comments (attached) in a point-by-point response. Submit the revised manuscript and response via the PLOS ONE submission system. Failure to adequately address these concerns may lead to further revisions or rejection. Contact the editorial office with any questions.

Sincerely,

[Note: HTML markup is below. Please do not edit.]

[NOTE: If reviewer comments were submitted as an attachment file, they will be attached to this email and accessible via the submission site. Please log into your account, locate the manuscript record, and check for the action link "View Attachments". If this link does not appear, there are no attachment files.]

While revising your submission, please upload your figure files to the Preflight Analysis and Conversion Engine (PACE) digital diagnostic tool, https://pacev2.apexcovantage.com/. PACE helps ensure that figures meet PLOS requirements. To use PACE, you must first register as a user. Registration is free. Then, login and navigate to the UPLOAD tab, where you will find detailed instructions on how to use the tool. If you encounter any issues or have any questions when using PACE, please email PLOS at figures@plos.org. Please note that Supporting Information files do not need this step.

# Author comment: Author response to Decision Letter 2

**DOI:** 10.1371/journal.pone.0352674.r004

15 Oct 2025

1. Comment: Clarity and Structure. The introduction and discussion lack focus. Clearly define the research gap and how your study addresses it. Restructure these sections to improve coherence and emphasize the study’s significance.

Response: In Introduction of the revision, the necessity of applying critical frequency in grammaticalization study is reinforced. It is carried out by the following steps: (1) limitations of the traditional frequency theory; (2) proposal of the critical frequency; (3) characteristics of the critical frequency; (4) measurement of the critical frequency; (5) significance of the critical frequency to grammaticalization study, especially the corpus-based study of grammaticalization.

In Discussion of the revision, the significance of subjectification to grammaticalization of be going to is highlighted. The study of the critical frequency can be taken as the integrating study of frequency and mechanism of grammaticalization. As Traugott (2010) put that subjectification is the subtype of the mechanism of syntactic change, the relationship between the grammaticalization of be going to and the subjectification of be going to needs further exploration.

2. Comment: Methodology. The experimental design and statistical methods need more detail and justification.

Response: In revision, the following parts are strengthened: (1) in Line 360-361, the application of Chi-square test is made clear; (2) in Line 395-399, the application of linear regression test is made clear; (3) in Line 404-408, the results from linear regression test are expounded.

3. Comment: Data Presentation. Figures and tables are unclear.

Response: In revision, the following parts are strengthened: (1) about Fig 1 and Table 1. It is made clear in the revision that the data in Fig 1 are from Table 1. And the data in Table 1 are explained in Line 273-275; (2) Example (17) and (18) are explained in Line 349-353; (3) Supporting Information Files describe the sources of the data and how they are obtained in Tables and Figs of the manuscript.

4. Comment: Literature Review. The manuscript requires a more thorough review of recent studies to better contextualize your findings within the field.

Response: In Line 67-77 of the revision, the studies of the grammaticalization of be going to are reviewed. The existing studies are categorized into 6 aspects. After identifying the limitations of existing studies, the necessity of this study is highlighted.

Attachment Submitted filename: Response_to_Reviewers_auresp_2.docx

# Editor report: Decision Letter 2

**DOI:** 10.1371/journal.pone.0352674.r005

**Authors:** Dawit Dibekulu

21 Oct 2025

PONE-D-25-26551R2

Revisiting the Grammaticalization of Future be going to: A Corpus-based Approach

PLOS ONE

Dear Dr. Dai,

Thank you for submitting your manuscript to PLOS ONE. After careful consideration, we feel that it has merit but does not fully meet PLOS ONE’s publication criteria as it currently stands. Therefore, we invite you to submit a revised version of the manuscript that addresses the points raised during the review process.

Please submit your revised manuscript by Dec 05 2025 11:59PM. If you will need more time than this to complete your revisions, please reply to this message or contact the journal office at plosone@plos.org. When you're ready to submit your revision, log on to https://www.editorialmanager.com/pone/ and select the 'Submissions Needing Revision' folder to locate your manuscript file.

Please include the following items when submitting your revised manuscript:

- A rebuttal letter that responds to each point raised by the academic editor and reviewer(s). You should upload this letter as a separate file labeled 'Response to Reviewers'.
- A marked-up copy of your manuscript that highlights changes made to the original version. You should upload this as a separate file labeled 'Revised Manuscript with Track Changes'.
- An unmarked version of your revised paper without tracked changes. You should upload this as a separate file labeled 'Manuscript'.

If you would like to make changes to your financial disclosure, please include your updated statement in your cover letter. Guidelines for resubmitting your figure files are available below the reviewer comments at the end of this letter.

If applicable, we recommend that you deposit your laboratory protocols in protocols.io to enhance the reproducibility of your results. Protocols.io assigns your protocol its own identifier (DOI) so that it can be cited independently in the future. For instructions see: https://journals.plos.org/plosone/s/submission-guidelines#loc-laboratory-protocols. Additionally, PLOS ONE offers an option for publishing peer-reviewed Lab Protocol articles, which describe protocols hosted on protocols.io. Read more information on sharing protocols at https://plos.org/protocols?utm_medium=editorial-email&utm_source=authorletters&utm_campaign=protocols.

We look forward to receiving your revised manuscript.

Kind regards,

Dawit Dibekulu, PhD

Academic Editor

PLOS ONE

Journal Requirements:

If the reviewer comments include a recommendation to cite specific previously published works, please review and evaluate these publications to determine whether they are relevant and should be cited. There is no requirement to cite these works unless the editor has indicated otherwise.

[Note: HTML markup is below. Please do not edit.]

[NOTE: If reviewer comments were submitted as an attachment file, they will be attached to this email and accessible via the submission site. Please log into your account, locate the manuscript record, and check for the action link "View Attachments". If this link does not appear, there are no attachment files.]

To ensure your figures meet our technical requirements, please review our figure guidelines: https://journals.plos.org/plosone/s/figures

You may also use PLOS’s free figure tool, NAAS, to help you prepare publication quality figures: https://journals.plos.org/plosone/s/figures#loc-tools-for-figure-preparation.

NAAS will assess whether your figures meet our technical requirements by comparing each figure against our figure specifications.

# Author comment: Author response to Decision Letter 3

**DOI:** 10.1371/journal.pone.0352674.r006

12 Dec 2025

We sincerely appreciate the editor and reviewers for their thoughtful feedback and constructive suggestions, which have greatly helped improve our manuscript. One-to-one responses to the comments and suggestions by the reviewers and editor are presented in the Rebuttal Letter.

Attachment Submitted filename: Response_to_Reviewers_auresp_3.docx

# Peer review: Decision Letter 3

**DOI:** 10.1371/journal.pone.0352674.r007

**Authors:** Dawit Dibekulu

2 Jan 2026

PONE-D-25-26551R3

Revisiting the Grammaticalization of Future be going to: A Corpus-based Approach

PLOS One

Dear Dr.  Dai,

Thank you for submitting your manuscript to PLOS ONE. After careful consideration, we feel that it has merit but does not fully meet PLOS ONE’s publication criteria as it currently stands. Therefore, we invite you to submit a revised version of the manuscript that addresses the points raised during the review process.

Please submit your revised manuscript by Feb 16 2026 11:59PM. If you will need more time than this to complete your revisions, please reply to this message or contact the journal office at plosone@plos.org. When you're ready to submit your revision, log on to https://www.editorialmanager.com/pone/ and select the 'Submissions Needing Revision' folder to locate your manuscript file.

Please include the following items when submitting your revised manuscript:

- A letter that responds to each point raised by the academic editor and reviewer(s). You should upload this letter as a separate file labeled 'Response to Reviewers'.
- A marked-up copy of your manuscript that highlights changes made to the original version. You should upload this as a separate file labeled 'Revised Manuscript with Track Changes'.
- An unmarked version of your revised paper without tracked changes. You should upload this as a separate file labeled 'Manuscript'.

If you would like to make changes to your financial disclosure, please include your updated statement in your cover letter. Guidelines for resubmitting your figure files are available below the reviewer comments at the end of this letter.

If applicable, we recommend that you deposit your laboratory protocols in protocols.io to enhance the reproducibility of your results. Protocols.io assigns your protocol its own identifier (DOI) so that it can be cited independently in the future. For instructions see: https://journals.plos.org/plosone/s/submission-guidelines#loc-laboratory-protocols. Additionally, PLOS ONE offers an option for publishing peer-reviewed Lab Protocol articles, which describe protocols hosted on protocols.io. Read more information on sharing protocols at https://plos.org/protocols?utm_medium=editorial-email&utm_source=authorletters&utm_campaign=protocols.

We look forward to receiving your revised manuscript.

Kind regards,

Dawit Dibekulu, PhD

Academic Editor

PLOS One

Journal Requirements:

1. If the reviewer comments include a recommendation to cite specific previously published works, please review and evaluate these publications to determine whether they are relevant and should be cited. There is no requirement to cite these works unless the editor has indicated otherwise.

[Note: HTML markup is below. Please do not edit.]

Reviewer's Responses to Questions

**Comments to the Author**

1. If the authors have adequately addressed your comments raised in a previous round of review and you feel that this manuscript is now acceptable for publication, you may indicate that here to bypass the “Comments to the Author” section, enter your conflict of interest statement in the “Confidential to Editor” section, and submit your "Accept" recommendation.

Reviewer #2: All comments have been addressed

Reviewer #3: All comments have been addressed

Reviewer #4: All comments have been addressed

**********

2. Is the manuscript technically sound, and do the data support the conclusions?

The manuscript must describe a technically sound piece of scientific research with data that supports the conclusions. Experiments must have been conducted rigorously, with appropriate controls, replication, and sample sizes. The conclusions must be drawn appropriately based on the data presented.

Reviewer #2: Yes

Reviewer #3: Yes

Reviewer #4: Partly

**********

3. Has the statistical analysis been performed appropriately and rigorously?

Reviewer #2: Yes

Reviewer #3: Yes

Reviewer #4: Yes

**********

4. Have the authors made all data underlying the findings in their manuscript fully available?

The PLOS Data policy requires authors to make all data underlying the findings described in their manuscript fully available without restriction, with rare exception (please refer to the Data Availability Statement in the manuscript PDF file). The data should be provided as part of the manuscript or its supporting information, or deposited to a public repository. For example, in addition to summary statistics, the data points behind means, medians and variance measures should be available. If there are restrictions on publicly sharing data—e.g. participant privacy or use of data from a third party—those must be specified.

Reviewer #2: Yes

Reviewer #3: Yes

Reviewer #4: Yes

**********

5. Is the manuscript presented in an intelligible fashion and written in standard English?

PLOS ONE does not copyedit accepted manuscripts, so the language in submitted articles must be clear, correct, and unambiguous. Any typographical or grammatical errors should be corrected at revision, so please note any specific errors here.

Reviewer #2: Yes

Reviewer #3: Yes

Reviewer #4: Yes

**********

6. Review Comments to the Author

Please use the space provided to explain your answers to the questions above. You may also include additional comments for the author, including concerns about dual publication, research ethics, or publication ethics. (Please upload your review as an attachment if it exceeds 20,000 characters)

Reviewer #2: The authors have adequately addressed all comments raised in the previous rounds of review. The manuscript is technically sound, and the corpus-based methodology is applied rigorously and transparently. The statistical analyses are appropriate for the research questions and are clearly reported, with results that support the conclusions drawn.

The use of large, well-established corpora and the clear operationalization of key concepts such as critical frequency, synchronic intensity, and diachronic thickness strengthen the reliability of the findings. Data availability complies with PLOS ONE requirements, as all relevant data are contained within the manuscript and its supporting information.

The manuscript is clearly written and well structured, and the argumentation is coherent throughout. I have no remaining concerns regarding research ethics, publication ethics, or dual publication. I consider the manuscript suitable for publication in its current form.

Reviewer #3: The abstract needs to be revisited to meet standard academic requirements. At present, it does not fully reflect the scope and contribution of the study. It is recommended that the abstract explicitly include the following elements:

• Research background/context (brief positioning within grammaticalization studies)

• Clear research objective(s)

• Data source(s) (type of corpus, size, period, or variety, where relevant)

• Methodological approach (corpus-based/quantitative–qualitative analysis)

• Key findings/results

• Main contribution/implications of the study

The revised abstract should be concise, informative, and reflective of what the study actually achieves.

Minor Comment

2. Methodology Section (Stylistic Adjustment)

In the methodology section, the use of future tense (e.g., “will be analyzed,” “will be examined”) should be avoided. Instead, the section should consistently employ:

• Past tense (to describe procedures already carried out), or

• Present simple tense (to describe standard methodological practices).

This change will improve academic style and align the manuscript with conventional research-writing norms.

Reviewer #4: General comments

1) Argumentation and Theoretical Balance The critique of frequency-based approaches to grammaticalization is persuasive, but the tone could be moderated slightly to acknowledge more explicitly what general frequency studies have successfully explained. Add more expressions in this regard

2)The staged examples are well chosen and clearly illustrate the proposed grammaticalization pathway. This is one of the manuscript’s strongest sections. However, some examples (e.g., highly marked or violent sentences) may raise stylistic concerns for certain journals; consider whether neutral alternatives could be substituted without loss of analytical value.

**********

7. PLOS authors have the option to publish the peer review history of their article (what does this mean?). If published, this will include your full peer review and any attached files.

If you choose “no”, your identity will remain anonymous but your review may still be made public.

**Do you want your identity to be public for this peer review?**  For information about this choice, including consent withdrawal, please see our Privacy Policy.

Reviewer #2: **Yes:** Mohanad Al Jbour

Reviewer #3: No

Reviewer #4: **Yes:** Aman Matebie (PhD)

**********

[NOTE: If reviewer comments were submitted as an attachment file, they will be attached to this email and accessible via the submission site. Please log into your account, locate the manuscript record, and check for the action link "View Attachments". If this link does not appear, there are no attachment files.]

To ensure your figures meet our technical requirements, please review our figure guidelines: https://journals.plos.org/plosone/s/figures

You may also use PLOS’s free figure tool, NAAS, to help you prepare publication quality figures: https://journals.plos.org/plosone/s/figures#loc-tools-for-figure-preparation.

NAAS will assess whether your figures meet our technical requirements by comparing each figure against our figure specifications.

Attachment Submitted filename: review report.docx

Attachment Submitted filename: Revisiting the grammaticalization of future be going to.docx

# Author comment: Author response to Decision Letter 4

**DOI:** 10.1371/journal.pone.0352674.r008

14 Feb 2026

We sincerely appreciate the editor and reviewers for their thoughtful feedback and constructive suggestions, which have greatly helped improve our manuscript. The following are one-to-one responses to the comments and suggestions.

Reviewers Comments

Reviewer 3

1. Comment: The abstract needs to be revisited to meet standard academic requirements.

Response: The revision gives full consideration of the suggestion, including research background, research objectives, data sources, methodological approach, key findings and main contribution of the study.

2. Comment: Stylistic Adjustment. In the methodology section, the section should consistently employ past tense or present simple tense.

Response: The revision employs the present simple tense in the methodology section to align the manuscript with conventional norms.

Reviewer 4

1. Comment: Argumentation and Theoretical Balance. “The tone could be moderated slightly to acknowledge more explicitly what general frequency studies have successfully explained.”

Response: The contribution of general frequency to grammaticalization is manifested in Line 59-61. The revision not only clarifies the significance of frequency to grammaticalization, but also highlights the quantitative research orientation, thus informing the methodological approach adopted in this study.

2. Comment: “some examples (e.g., highly marked or violent sentences) may raise stylistic concerns for certain journals; consider whether neutral alternatives could be substituted without loss of analytical value.”

Response: In Line 210 and Line 214, example (7) and (10) are substituted to avoid any possible stylistic concerns.

Attachment Submitted filename: Response_to_Reviewers_auresp_4.docx

# Peer review: Decision Letter 4

**DOI:** 10.1371/journal.pone.0352674.r009

**Authors:** Dawit Dibekulu

5 Apr 2026

PONE-D-25-26551R4

Revisiting the Grammaticalization of Future be going to: A Corpus-based Approach

PLOS One

Dear Dr. Dai,

Thank you for submitting your manuscript to PLOS ONE. After careful consideration, we feel that it has merit but does not fully meet PLOS ONE’s publication criteria as it currently stands. Therefore, we invite you to submit a revised version of the manuscript that addresses the points raised during the review process.

Please submit your revised manuscript by May 20 2026 11:59PM. If you will need more time than this to complete your revisions, please reply to this message or contact the journal office at plosone@plos.org. When you're ready to submit your revision, log on to https://www.editorialmanager.com/pone/ and select the 'Submissions Needing Revision' folder to locate your manuscript file.

Please include the following items when submitting your revised manuscript:

- A letter that responds to each point raised by the academic editor and reviewer(s). You should upload this letter as a separate file labeled 'Response to Reviewers'.
- A marked-up copy of your manuscript that highlights changes made to the original version. You should upload this as a separate file labeled 'Revised Manuscript with Track Changes'.
- An unmarked version of your revised paper without tracked changes. You should upload this as a separate file labeled 'Manuscript'.

If you would like to make changes to your financial disclosure, please include your updated statement in your cover letter. Guidelines for resubmitting your figure files are available below the reviewer comments at the end of this letter.

If applicable, we recommend that you deposit your laboratory protocols in protocols.io to enhance the reproducibility of your results. Protocols.io assigns your protocol its own identifier (DOI) so that it can be cited independently in the future. For instructions see: https://journals.plos.org/plosone/s/submission-guidelines#loc-laboratory-protocols. Additionally, PLOS ONE offers an option for publishing peer-reviewed Lab Protocol articles, which describe protocols hosted on protocols.io. Read more information on sharing protocols at https://plos.org/protocols?utm_medium=editorial-email&utm_source=authorletters&utm_campaign=protocols.

As the corresponding author, your ORCID iD is verified in the submission system and will appear in the published article. PLOS supports the use of ORCID, and we encourage all coauthors to register for an ORCID iD and use it as well. Please encourage your coauthors to verify their ORCID iD within the submission system before final acceptance, as unverified ORCID iDs will not appear in the published article. *Only*  the individual author can complete the verification step; PLOS staff *cannot*  verify ORCID iDs on behalf of authors.

We look forward to receiving your revised manuscript.

Kind regards,

Dawit Dibekulu, PhD

Academic Editor

PLOS One

Journal Requirements:

1. If the reviewer comments include a recommendation to cite specific previously published works, please review and evaluate these publications to determine whether they are relevant and should be cited. There is no requirement to cite these works unless the editor has indicated otherwise.

2. Please review your reference list to ensure that it is complete and correct. If you have cited papers that have been retracted, please include the rationale for doing so in the manuscript text, or remove these references and replace them with relevant current references. Any changes to the reference list should be mentioned in the rebuttal letter that accompanies your revised manuscript. If you need to cite a retracted article, indicate the article’s retracted status in the References list and also include a citation and full reference for the retraction notice.

[Note: HTML markup is below. Please do not edit.]

Reviewers' comments:

Reviewer's Responses to Questions

**Comments to the Author**

1. If the authors have adequately addressed your comments raised in a previous round of review and you feel that this manuscript is now acceptable for publication, you may indicate that here to bypass the “Comments to the Author” section, enter your conflict of interest statement in the “Confidential to Editor” section, and submit your "Accept" recommendation.

Reviewer #3: All comments have been addressed

Reviewer #4: All comments have been addressed

**********

2. Is the manuscript technically sound, and do the data support the conclusions?

The manuscript must describe a technically sound piece of scientific research with data that supports the conclusions. Experiments must have been conducted rigorously, with appropriate controls, replication, and sample sizes. The conclusions must be drawn appropriately based on the data presented.

Reviewer #3: Yes

Reviewer #4: Yes

**********

3. Has the statistical analysis been performed appropriately and rigorously?

Reviewer #3: (No Response)

Reviewer #4: Yes

**********

4. Have the authors made all data underlying the findings in their manuscript fully available?

The PLOS Data policy requires authors to make all data underlying the findings described in their manuscript fully available without restriction, with rare exception (please refer to the Data Availability Statement in the manuscript PDF file). The data should be provided as part of the manuscript or its supporting information, or deposited to a public repository. For example, in addition to summary statistics, the data points behind means, medians and variance measures should be available. If there are restrictions on publicly sharing data—e.g. participant privacy or use of data from a third party—those must be specified.

Reviewer #3: Yes

Reviewer #4: Yes

**********

5. Is the manuscript presented in an intelligible fashion and written in standard English?

PLOS ONE does not copyedit accepted manuscripts, so the language in submitted articles must be clear, correct, and unambiguous. Any typographical or grammatical errors should be corrected at revision, so please note any specific errors here.

Reviewer #3: Yes

Reviewer #4: Yes

**********

6. Review Comments to the Author

Please use the space provided to explain your answers to the questions above. You may also include additional comments for the author, including concerns about dual publication, research ethics, or publication ethics. (Please upload your review as an attachment if it exceeds 20,000 characters)

Reviewer #3: Recommendation

•The paper is overly long and occasionally repetitive:

•The literature review could be streamlined

•The distinction between critical frequency and general frequency is asserted but not rigorously justified.

•The criteria for identifying “critical contexts” are not replicable:

•The classification of verbs into three types appears ad hoc.

•No clear annotation protocol or inter-rater reliability is provided.

•The leap from correlation to “threshold triggering grammaticalization” is theoretically overstated.

•No validation or manual checking procedures are reported.

•Improve statistical rigor and interpretation

•Substantially revise language and structure

Reviewer #4: The article is thoroughly written and presents obvious better structure and presentation. The introduction is relevant and offers the proper background and leads to the research objective successfully, whereas the statement of the problem is well-defined and applicable. The methodology section is presented in a logical way that enables the reader to know the approach that was employed, and the analysis section is clearly structured with the results that substantiate the discussion as a whole. In general, the manuscript is appropriate to the context of the journal and of high academic standard.

**********

7. PLOS authors have the option to publish the peer review history of their article (what does this mean?). If published, this will include your full peer review and any attached files.

If you choose “no”, your identity will remain anonymous but your review may still be made public.

**Do you want your identity to be public for this peer review?**  For information about this choice, including consent withdrawal, please see our Privacy Policy.

Reviewer #3: **Yes:** Muhammad Ajmal

Reviewer #4: **Yes:** Aman Matebie (PhD)

**********

[NOTE: If reviewer comments were submitted as an attachment file, they will be attached to this email and accessible via the submission site. Please log into your account, locate the manuscript record, and check for the action link "View Attachments". If this link does not appear, there are no attachment files.]

To ensure your figures meet our technical requirements, please review our figure guidelines: https://journals.plos.org/plosone/s/figures

You may also use PLOS’s free figure tool, NAAS, to help you prepare publication quality figures: https://journals.plos.org/plosone/s/figures#loc-tools-for-figure-preparation.

NAAS will assess whether your figures meet our technical requirements by comparing each figure against our figure specifications.

# Author comment: Author response to Decision Letter 5

**DOI:** 10.1371/journal.pone.0352674.r010

8 Jun 2026

We sincerely appreciate the editor and reviewers for their thoughtful feedback and constructive suggestions, which have greatly helped improve our manuscript. The following are one-to-one responses to the comments and suggestions.

Reviewers Comments

Comment: The paper is overly long and occasionally repetitive.

Response:

We thank the reviewer for this constructive comment. We agree that the original manuscript was unnecessarily lengthy and contained some redundancy. In response, we have substantially shortened and streamlined the manuscript.

Specifically, we have made the following changes:

(1) The detailed corpora descriptions have been removed from the Abstract, as this information is already fully presented in the Methodology section.

(2) Throughout the paper, identical or similar expressions have been integrated and streamlined to avoid repetition and enhance conciseness, as will be shown in the next Comment and Response.

We believe the revised manuscript is now more concise, readable, and free of unnecessary repetition, while retaining all essential information and methodological transparency. We hope that the revised version now meets the journal’s standards for length and clarity.

Comment: The literature review could be streamlined.

Response:

We thank the reviewer for this helpful suggestion. We agree that the literature review in the original submission was longer than necessary and included some repetitive references. In response, we have thoroughly revised this section to make it more focused, concise, and directly relevant to the study’s objectives.

Specifically, we have removed references that provided only general background or were only loosely related to our specific research question. For example:

(1) In the previous draft, the premise of grammaticalization by Peng [21] appeared in both the “Context” section and the “Critical Context and Critical Frequency” section. In the current draft, this reference has been retained only in the latter to eliminate redundancy and focus on the specific topic.

(2) Similarly, Critical Frequency Hypothesis by Peng [21] (i.e., which posits that providing the critical frequency accumulates a considerable diachronic thickness and reaches a certain synchronic intensity, grammaticalization may be triggered.) was previously cited in both the Introduction and the literature review. In the present draft, it has been removed from the literature review while remaining in the Introduction.

We believe these revisions have resulted in a more streamlined literature review that is easier to follow and better aligned with the journal’s expectations for concise yet comprehensive background information. All essential prior work remains appropriately cited, and the logical link to our study’s rationale is now clearer.

Comment: The distinction between critical frequency and general frequency is asserted but not rigorously justified.

Response:

We thank the reviewer for this insightful comment. In the original manuscript, the distinction between critical frequency and general frequency may not have been sufficiently justified. To address this concern, we have now provided a more rigorous rationale from the following aspects:

(1) Theoretically, not all frequencies observed during the grammaticalization process of a given grammaticalizing item can trigger grammaticalization. Heine [19] and Diewald [20] concurrently introduced the concept of “critical context”, in which the frequencies observed are accordingly termed “critical frequencies”. Peng [21] further distinguished two types of frequencies of the grammaticalizing items, i.e., critical frequency and non-critical frequency. Critical frequency is independent of general frequency, in that an increase of the former may (but not necessarily) accompany an increase of the latter.

(2) Empirically, Peng [21] quantitatively investigated the critical frequency of selected morphemes and constructions in Chinese by the application of corpora. Peng [35] further explored two issues concerning critical frequency: (1) whether there exists a positive correlation between general frequency and critical frequency; (2) what the minimum critical frequency required for pragmatic implicature to become conventionalized is.

(3) In the present paper, we focus our research on two issues: ① the critical frequency of future be going to; ② the relationship between its critical frequency and general frequency. Since both issues have been exhaustively examined in the manuscript, we will not reiterate the detailed findings here.

We believe this expounded rationale clarifies the theoretical and empirical basis for distinguishing critical frequency from general frequency and properly situates our research within the existing literature.

Comment: The criteria for identifying “critical contexts” are not replicable.

Response:

We thank the reviewer for raising this important point regarding replicability. We agree that, in principle, replicability may not be an inherent or necessary attribute of critical contexts across all cases, given that different morphemes or constructions may involve distinct critical contexts, and any case that triggers grammaticalization may be considered valid. In fact, as mentioned in our paper, “A linguistic form may be associated with a number of different critical contexts”. Nevertheless, for the specific case of be going to, we have taken concrete steps to ensure replicability. Specifically, we formalized the critical context through explicit logical reasoning and conducted an exhaustive corpus retrieval, which enabled us to determine its critical frequency in an objective and transparent manner. Consequently, the identification of the critical context for the grammaticalization of be going to in our study is fully replicable.

Comment: The classification of verbs into three types appears ad hoc.

Response:

We thank the reviewer for this comment. We would like to clarify that the threefold classification of verbs in our study is not ad hoc but is systematically derived from the three distinct contexts involved in the grammaticalization of be going to, namely the initial stage, the critical context, and the isolating context (see Lines 117–134).

In the initial stage, grammaticalization is not yet triggered. Here, be going to exclusively denotes movement in space and therefore co-occurs only with action verbs. In the critical context, ambiguity arises, allowing be going to to refer either to spatial movement or future time. In the isolating context, as noted in Line 128, “the isolating context is incompatible with some salient properties of the source meaning”; consequently, be going to refers unambiguously to future time and can be followed by mental or stative verbs.

Correspondingly, verbs following future be going to naturally fall into three categories:

(1) Verbs in the initial stage. These are action verbs that occur in both go and V and go to V constructions and do not cause ambiguity. They are empirically retrievable from corpora.

(2) Verbs in the isolating context. These are mental or stative verbs. They are the indicator of the full grammaticalization of be going to. The identification of this type of words follows the well-established criteria of Halliday & Matthiessen [39].

(3) Verbs in the critical context. These verbs have the potential to trigger grammaticalization of be going to. They are derived by subtracting the first two categories from the total set of verbs attested during the grammaticalization process of be going to.

The complete procedure for obtaining these three categories is detailed in Lines 391–401 of the revised manuscript.

Methodologically, all the verbs appearing during the whole grammaticalization process of future be going to have been exhaustively retrieved, with no exceptions. Theoretically, the identification of the 159 words is strictly justified with reference to well-established or independently verified criteria, including the grammaticalization process of go and V to go to V, as well as the framework proposed by Halliday & Matthiessen [39].

Therefore, far from being ad hoc, our classification is grounded in both rigorous methodology and sound theoretical justification.

Comment: No clear annotation protocol or inter-rater reliability is provided.

Response:

Thank you for this comment. We would like to clarify that the present study does not involve any manual annotation or subjective classification by human coders. Therefore, an annotation protocol or inter-rater reliability (IRR) is not applicable to our methodology.

Specifically, all data in this study are obtained through the following approaches:

(1) Exhaustive corpus retrieval using formalized search queries based on explicit linguistic criteria. The study is built on large, balanced, and authoritative diachronic corpora, with corpus sizes reported in the manuscript. Exhaustive retrieval—rather than random sampling—was performed using predefined search queries, ensuring the replicability of both the retrieved data and the results.

(2) Logical determination of critical contexts, as illustrated by the grammaticalization of be going to. With ambiguity as its distinctive features, the critical context is rigorously defined (including the procedure for identifying the 159 words). The critical frequency is derived through logical reasoning, independent of any subjective judgment or inter-rater input. The formalization of the critical context and the formulation of search queries remain consistent regardless of the researcher involved.

All retrieved data are presented as standardized frequencies per million words to ensure comparability across texts of different sizes or from different historical periods.

Since no manual annotation or subjective classification was performed—i.e., the preconditions for requiring an annotation protocol and IRR are absent— we respectfully maintain that such procedures are not necessary for this study. We hope this clarification addresses the concern. Thank you again for your careful reading.

Comment: The leap from correlation to “threshold triggering grammaticalization” is theoretically overstated.

Response:

Thank you for this constructive comment. We acknowledge that our use of the so-called “threshold triggering” may inadvertently imply a stronger causal claim than intended. In response, we have revised our interpretation as follows:

(1) The concept of “critical frequency,” operationalized in this study as diachronic thickness and synchronic intensity, is a central focus of our investigation into the grammaticalization of be going to. We suggest that it is critical frequency, rather than general frequency, that plays a more direct role in facilitating grammaticalization. In this sense, critical frequency can be seen as a driving force behind the process. The term “threshold” is used heuristically and metaphorically to suggest that grammaticalization may require a certain level of critical frequency to take effect.

(2) The procedures for determining diachronic thickness and synchronic intensity are thoroughly and rigorously detailed in the present study.

(3) Additionally, a linear regression test was conducted to examine the relationship between general frequency and critical frequency. The results show a strong correlation between the two for be going to.

(4) Given this correlation, we do not claim a direct causal leap from correlation to the proposed “threshold triggering.” Instead, we treat the threshold metaphor as a descriptive heuristic for understanding how critical frequency may contribute to grammaticalization, rather than positing a discrete triggering event.

We hope that these have adequately addressed the concern by clarifying the theoretical status of “threshold” and avoiding any overstatement of causality.

Comment: No validation or manual checking procedures are reported.

Response:

We thank the reviewer for raising this important point. We agree that validation and manual checking are essential, even for exhaustive corpus queries. In the original manuscript, we did not report such procedures, as this study did not employ statistical models (e.g., classifiers) that require manually annotated training data. However, in response to the reviewer’s comment, we have conducted a post-hoc manual validation.

(1) Verification criteria: All 159 retrieved instances were manually examined against the predefined linguistic criteria, such as that by Halliday & Matthiessen [39]. Specifically, we verified whether each instance truly conforms to the formalized definition of the target category (e.g., the critical context of be going to).

(2) Accuracy assessment: The number of correctly retrieved instances was counted, and the accuracy rate was calculated as [correct instances / total instances × 100%]. The observed accuracy was 99.37%, indicating that our search queries and retrieval procedures are highly reliable.

This manual checking confirms that the vast majority of the retrieved data meet the established criteria, and the results reported in this study are robust. We hope this addresses the reviewer’s concern.

Comment: Improve statistical rigor and interpretation.

Response:

We thank the reviewer for this valuable suggestion. We have improved the statistical rigor and interpretation of our results accordingly. Specifically, we have clarified the linear regression analysis examining the relationship between critical frequency (as the independent variable, x) and general frequency (as the dependent variable, y).

(1) The regression model reveals a strong positive linear relationship between x and y, with the fitted equation y=10.046x+0.08.

(2) The coefficient of determination R^2=0.984 indicates that 98.4% of the variance in y is accounted for by x. This high value reflects an excellent model fit and suggests that the two frequency measures are very strongly associated.

(3) The slope (10.046) indicates a directly proportional relationship: a one-unit increase in x corresponds to an increase of approximately 10.046 units in y. The intercept (0.08) estimates the value of y when x is zero.

We emphasize that this regression analysis is intended to quantify the strength of the linear association between the two frequency measures, rather than to claim a causal direction. These results have been clearly described in the revised manuscript (see Lines 412—418). We believe these revisions address the reviewer’s concern regarding statistical rigor and interpretation. Thank you for your careful reading.

Comment: Substantially revise language and structure.

Response:

We have substantially revised the language and structure throughout the manuscript in response to this comment. The revision was carried out by the author in collaboration with professional academic editors to ensure idiomaticity and overall readability. To provide a concrete example, the paragraph originally located in Lines 250—253 has been restructured in Lines 174—177 to achieve greater logical fluency.

We hope that the revised manuscript now meets the language standards expected by PLOS ONE.

Attachment Submitted filename: Response_to_Reviewers_auresp_5.docx

# Editor report: Decision Letter 5

**DOI:** 10.1371/journal.pone.0352674.r011

**Authors:** Dawit Dibekulu

14 Jun 2026

Revisiting the Grammaticalization of Future be going to: A Corpus-based Approach

PONE-D-25-26551R5

Dear Dr. Dai,

We’re pleased to inform you that your manuscript has been judged scientifically suitable for publication and will be formally accepted for publication once it meets all outstanding technical requirements.

Within one week, you’ll receive an e-mail detailing the required amendments. When these have been addressed, you’ll receive a formal acceptance letter and your manuscript will be scheduled for publication.

An invoice will be generated when your article is formally accepted. Please note, if your institution has a publishing partnership with PLOS and your article meets the relevant criteria, all or part of your publication costs will be covered. Please make sure your user information is up-to-date by logging into Editorial Manager at Editorial Manager® and clicking the ‘Update My Information' link at the top of the page. For questions related to billing, please contact billing support.

If your institution or institutions have a press office, please notify them about your upcoming paper to help maximize its impact. If they’ll be preparing press materials, please inform our press team as soon as possible -- no later than 48 hours after receiving the formal acceptance. Your manuscript will remain under strict press embargo until 2 pm Eastern Time on the date of publication. For more information, please contact onepress@plos.org.

Kind regards,

Dawit Dibekulu, PhD

Academic Editor

PLOS One

Additional Editor Comments (optional):

Reviewers' comments:

# Editor report: Acceptance letter

**DOI:** 10.1371/journal.pone.0352674.r012

**Authors:** Dawit Dibekulu

PONE-D-25-26551R5

PLOS One

Dear Dr. Dai,

I'm pleased to inform you that your manuscript has been deemed suitable for publication in PLOS One. Congratulations! Your manuscript is now being handed over to our production team.

At this stage, our production department will prepare your paper for publication. This includes ensuring the following:

* All references, tables, and figures are properly cited

* All relevant supporting information is included in the manuscript submission,

* There are no issues that prevent the paper from being properly typeset

You will receive further instructions from the production team, including instructions on how to review your proof when it is ready. Please keep in mind that we are working through a large volume of accepted articles, so please give us a few days to review your paper and let you know the next and final steps.

Lastly, if your institution or institutions have a press office, please let them know about your upcoming paper now to help maximize its impact. If they'll be preparing press materials, please inform our press team within the next 48 hours. Your manuscript will remain under strict press embargo until 2 pm Eastern Time on the date of publication. For more information, please contact onepress@plos.org.

You will receive an invoice from PLOS for your publication fee after your manuscript has reached the completed accept phase. If you receive an email requesting payment before acceptance or for any other service, this may be a phishing scheme. Learn how to identify phishing emails and protect your accounts at https://explore.plos.org/phishing.

If we can help with anything else, please email us at customercare@plos.org.

Thank you for submitting your work to PLOS ONE and supporting open access.

Kind regards,

PLOS ONE Editorial Office Staff

on behalf of

Dr. Dawit Dibekulu

Academic Editor

PLOS One
